# The therapeutic potential of low-intensity focused ultrasound for treating substance use disorder

**DOI:** 10.3389/fpsyt.2024.1466506

**Published:** 2024-11-19

**Authors:** Greatness O. Olaitan, Wendy J. Lynch, B. Jill Venton

**Affiliations:** ^1^ Department of Chemistry, University of Virginia, Charlottesville, VA, United States; ^2^ Psychiatry and Neurobehavioral Sciences, University of Virginia, Charlottesville, VA, United States

**Keywords:** substance use disorder, low-intensity focused ultrasound, neuromodulation, neurotherapeutics, psychiatry

## Abstract

Substance use disorder (SUD) is a persistent public health issue that necessitates the exploration of novel therapeutic interventions. Low-intensity focused ultrasound (LIFU) is a promising modality for precise and invasive modulation of brain activity, capable of redefining the landscape of SUD treatment. The review overviews effective LIFU neuromodulatory parameters and molecular mechanisms, focusing on the modulation of reward pathways in key brain regions in animal and human models. Integration of LIFU with established therapeutics holds promise for augmenting treatment outcomes in SUD. The current research examines LIFU’s efficacy in reducing cravings and withdrawal symptoms. LIFU shows promise for reducing cravings, modulating reward circuitry, and addressing interoceptive dysregulation and emotional distress. Selecting optimal parameters, encompassing frequency, burst patterns, and intensity, is pivotal for balancing therapeutic efficacy and safety. However, inconsistencies in empirical findings warrant further research on optimal treatment parameters, physiological action mechanisms, and long-term effects. Collaborative interdisciplinary investigations are imperative to fully realize LIFU’s potential in revolutionizing SUD treatment paradigms and enhancing patient outcomes.

## 1 Introduction

Substance use disorder (SUD) persists as one of the most challenging public health issues, with millions globally struggling with the devasting effects of the disease. The escalating numbers, as reported by the United Nations Office on Drugs and Crime (UNODC), signal a growing epidemic, from 27 million people having a SUD in 2016 to 46 million people in 2022 (1–3). Key contributors to SUD prevalence include opioids, psychostimulants, and alcohol (4). Opioids, particularly fentanyl, are the primary drivers of the overdose epidemic, resulting in over 100,000 deaths annually in the US alone (5). Psychostimulants rank as the second leading cause of overdose deaths, yet there is no FDA-approved medication for treating psychostimulant use disorder (6). Alcohol use disorder remains one of the most widespread and devastating public health issues, with millions affected globally (7, 8). SUD’s immense societal impact and costs highlight the urgent need for effective treatments. This need is especially critical for addressing the challenges posed by psychostimulants and opioids. The lack of approved treatments for psychostimulant use disorder and the limitations and stigma associated with existing opioid SUD treatments necessitate the development of novel interventions (9). Addressing SUD demands immediate attention to developing more accessible and effective treatment options.

Developing effective pharmacological treatments for SUD is challenging due to the diverse action mechanisms of addictive drugs, each inducing varied neuromodulatory and addictive effects (10). Despite meticulous drug design informed by understanding these distinct mechanisms, today’s best treatment regimen results in persistent craving and relapse in 40-60%, suggesting the involvement of multiple brain circuits in craving and relapse (11). Pharmacological interventions target specific drug action mechanisms, while non-pharmacological approaches address circuits activated by different drugs based on their primary action (12). However, there is a growing consensus on the role of common pathways in SUD, particularly those involving dysregulation of dopamine signaling within the reward pathway (13), and the progressive recruitment of glutamatergic signaling and other brain regions (amygdala, insular cortex), leading to withdrawal symptoms, negative affect, compulsive drug use, and strong urges to obtain the drug at all costs (14, 15). Human and animal studies point to a shared neurobiological foundation for relapse, highlighting the crucial role of glutamatergic projections from the PFC to the nucleus accumbens (NAc) (16, 17), making interventions targeting these disrupted circuits promising for SUD management (18, 19). Neuromodulation, mainly through noninvasive techniques like transcranial direct current stimulation (tDCS) and transcranial magnetic stimulation (TMS), safely modulate cortical and subcortical functions, has shown promise in this regard by effectively reducing drug craving in individuals with SUD (20). However, these methods face limitations related to penetration depth and spatial resolution (21, 22).

Low-Intensity Focused Ultrasound (LIFU) emerges as a novel neuromodulation strategy, offering targeted intervention for neural circuits affected by SUD (23). LIFU’s precise, noninvasive modulation of brain activity, high spatial resolution, and deep penetration make it a promising candidate for improving treatment outcomes in SUD. Despite the challenges in measuring its effects and fully understanding its mechanisms (24), the potential of LIFU for SUD treatment is significant as it can both excite or inhibit neuronal activity (25).

In this review, we will explore LIFU’s potential in the context of SUD treatment, emphasizing the neurobiological aspects of SUD to opioids, psychostimulants, and alcohol. We begin by reviewing brain circuits critical for the reward pathway, the craving/relapse circuitry, and the interoception circuitry. Then, we explore the advantages of LIFU over other neuromodulatory methods, assess typical readouts for LIFU’s actions, and discuss the physiological mechanisms of LIFU’s effects. Subsequently, we survey the neuromodulation parameters of LIFU that are essential for inducing excitatory or inhibitory effects. We then synthesize and present the findings from prior studies investigating LIFU’s efficacy in relevant animal models and humans with SUD.

## 2 Brain circuitry and neurotransmission in addiction

The brain circuit pathways for SUD are similar, although different drugs may target distinct receptors or proteins (12). A dynamic interplay between the reward pathway (reward-related behavior), the craving/relapse circuitry (driving drug-seeking behavior), and the interoceptive system (processing internal signals) intricately links craving and compulsion (26–28). The disruption within these pathways and the intricate communication between them are central to addictive behavior and physiological effects during SUD and withdrawal (29). These disruptions are shown in changes in neurotransmission, neuroactivity (neuronal firing and oscillation frequency), and neuron—or drug-specific adaptation gene expression, potentially leading to drug-seeking or craving reinforcement.

### 2.1 Connectivity, functioning, and drug adaptation in the reward and craving/relapse circuitry

While the reward system primarily motivates the initial drug use by reinforcing pleasure, the craving/relapse circuitry sustains SUD by driving compulsive drug-seeking behavior. Changes in memory and emotional processing systems increase vulnerability to relapse. Although the Nucleus Accumbens (NAc) is the central hub of the two systems, the Amygdala (AMY) regulates emotional processing, and the Hippocampus (HP) functions in memory processing (30, 31). The Prefrontal cortex (PFC), on the other hand, regulates the function of the three regions (32, 33).

The NAc, divided into core and shell regions, receives inputs from the ventral tegmental area (VTA), AMY, and HP and sends outputs to the PFC (34–36). The core differs from the shell’s inputs, with the former receiving from the central AMY nucleus and PFC and the latter from the lateral hypothalamus (LH) (37–42). Both regions are involved in reward behaviors, but the core shows drug-specific adaptations, suggesting potential for SUD treatments, though further research is needed (43–45). Notably, the dorsomedial PFC (dmPFC), a crucial regulator of cravings and drug-seeking behavior, can become compromised in SUD (46, 47). Within the dmPFC, the prelimbic (PLC) cortex is more implicated in relapse susceptibility, while the infralimbic (ILC) cortex potentially aids in suppressing drug-seeking behavior (46, 47). This region’s activity evolves during withdrawal, initially showing hypoactivity in early withdrawal but hyperactivity in late withdrawal (48–51). Similarly, the amygdala undergoes structural changes and adaptations from chronic drug use, amplifying circuits linked to craving and relapse (26, 52). Imbalances in neurotransmitters within the amygdala can significantly alter how reward signals are processed, thus contributing to addictive behaviors (53–57). Different subdivisions of the AMY respond differently to drug versus natural rewards. While the basolateral AMY (BLA) is involved in both, the central AMY (CeA) primarily mediates drug-reward-driven behaviors (55–57). While the HP is not typically a primary target in treating SUD, disruption in HP theta oscillations could occur in SUD, leading to imbalances in neurotransmission in connected brain regions, including the AMY, HP, and PFC.

Addictive drugs, such as psychostimulants, opioids, and alcohol, change neurotransmission in both craving and reward systems, albeit with varying mechanisms and effects (58–61). For example, exposure to psychostimulants can lead to dysfunction in the glutamatergic projections between the dmPFC and NAc while affecting NAc dopamine by dopamine transporter blockage, eventually leading to drug-seeking reinforcement (62–65). Conversely, the amygdala’s response to psychostimulants is expressed by surges in dopamine activity (66–68). Opioids engage opioid receptors, inducing euphoria, pain relief, and dopamine release while affecting glutamate and dopamine transmission (69). Alcohol increases BLA glutamate, CeA GABA, and NAc core dopamine, fostering intoxication, reward perception, emotional reactions, and impulsivity (70–72). Chronic psychostimulants and opioid use can heighten craving, desensitize natural reward responses, and induce enduring neurological alterations, leading to adaptations, tolerance, and a shift from positive to negative reinforcement effects (73–75). In the same way, prolonged alcohol use can disrupt PFC glutamate signaling, impairing cognitive faculties and decision-making (76). Therefore, neuromodulation of the reward and craving/relapse systems is crucial for developing targeted interventions for disrupting SUD-related cycles and promoting recovery.

### 2.2 Connectivity, functioning, and drug adaptation in the interoceptive circuitry

The Insular cortex (IC), a vital component of the interoceptive system, works alongside the ACC in processing internal bodily signals and drug cravings (77). Functionally divided into ventral, dorsal anterior, and posterior regions, the IC retrieves memories of drug effects stored in the ventral anterior IC (AIC) during drug craving, triggering the posterior IC (PIC) to process physical sensations associated with the drug (78–80). Collaborating with the HP, PFC, and AMY, the IC amplifies emotions during drug withdrawal, with PFC activity varying based on the withdrawal stage (78–81). This collaboration intensifies the conscious experience of withdrawal symptoms.

The IC’s adaptation to drug versus natural rewards is complex, involving local communication within the ventral anterior IC to regulate activity and balance glutamate influence (79). Further research is needed to compare IC adaptation to drugs versus natural rewards. In the PFC, ACC processes information, integrating physiological signals about the body’s internal state and assigns salience to these signals, influencing perception and behavior (82). The ACC, interacting with the IC, modulates attention, decision-making, and emotional regulation, driving motivation to seek relief from drug use (83, 84). However, drug memories can override its guidance, leading to relapse (80, 85).

Psychostimulants, opioids, and alcohol disrupt the interoceptive system’s delicate equilibrium via neurotransmitter modulation, obscure overdose symptoms, and foster hazardous behaviors (86). Chronic substance use can precipitate enduring modifications in interoceptive processing, reinforcing drug use as a coping mechanism (87). This intricate network interaction, termed allostasis dysregulation, intensifies craving and compulsive drug use in individuals with SUD, often manifesting as withdrawal symptoms and recurrent substance-seeking behavior (88, 89). Thus, the interoceptive system is an important target to disrupt the craving-compulsion cycle and address SUD effectively.

## 3 LIFU as a neuromodulatory intervention

### 3.1 LIFU versus other noninvasive neuromodulation techniques

Numerous techniques have been developed to treat psychological disorders by facilitating neuromodulation (90). Traditional methods like TMS and TDCS have provided valuable insights into noninvasive strategies for altering brain function and treating disease. Treatments with these methods are relatively painless, do not require surgery, and can be combined with other therapies (20). However, their effects can vary, and they have limited spatial resolution to target specific brain regions (20, 21). Emerging technologies, such as auricular nerve stimulation and near-infrared optogenetic stimulation (NIR), address some of these challenges but face their limitations (see **Table 1** for a summary of the advantages and limitations of various neuromodulatory technologies).

**Table 1:** Strengths and weaknesses of various non/minimally invasive neuromodulatory techniques.

Technique	Energy Type	Resolution	Penetration	Strengths	Weakness and Considerations
**Transcranial Magnetic Stimulation (TMS)**	Magnetic	3-5 cm	1-3 cm	• Established efficacy	• Limited resolution and penetration due to absorption and scattering • Discomfort/tingling at the site of stimulation
**Nanomaterial-Enabled Magnetic Stimulation (NEMS)**	Magneto-thermal, -electrical, -mechanical	≥ 1 nm	Unlimited in theory*	• NEMS Allows for selective targeting of neurons/circuits with high resolution. • NEMS does not require genetic engineering. • Long-term effectiveness.	• NEMS requires invasive magnetic nanoparticle (MNP) injection. • Scaling magnetic coils to deep brain regions in humans poses a significant challenge. • Heating of MNPs may result in aggregation, brain swelling, and increased intracranial pressure. • long-term toxicological effects and clearance of MNPs • Evidence of efficacy is based predominantly on small animal models
**Transcranial Direct Current Stimulation (TDCS)**	Electrical	≥ 0.5 cm	1-2 cm	• Established efficacy	• Pain at the site of stimulation limits the magnitude of modulation. • Limited resolution and penetration due to tissue conductivity.
**Auricular nerve stimulation (ANS)**	Electrical	1-10 mm	0.5-10 mm	• It has a wide range of applications as a peripheral nervous system stimulator.	• ANS is considered a complementary therapy, not a stand-alone treatment. • ANS does not directly impact the brain
**Temporal Interference Stimulation (TIS)**	Electrical	≥2 mm	5 cm	• TIS can mitigate the inadvertent stimulation of scalp nerves and concomitant scalp discomfort. • Could use established protocols from deep brain stimulation and TDCS	• Low spatial resolution • Selectively targeting deep, small brain structures may not be possible. • Specific positioning schemes for target brain regions are currently unavailable. • Clinical trials are needed.
**Nanoparticle-coupled Near-Infrared Optogenetic** **Stimulation (NIR)**	Near-infrared	≥ 10 µm	≤1 cm	• NIR allows for selective targeting of neurons/circuits with high resolution.	• NIR requires invasive nanoparticle/viral vector injection. • Effective delivery and long-term safety of viral vectors used for genetic modification poses a challenge. • Unique nanostructures are required for each new application. • Near-infrared light struggles to reach very deep brain regions. • Evidence-based predominantly on small animal models
**Low-intensity focused ultrasound (LIFU)**	Mechanical	1-3 mm	10-15 cm	• LIFU allows for selective targeting of circuits and small brain regions with high resolution. • It can target deep brain regions. • Established safety. • LIFU offers compatibility with nanoparticles, microbubbles, and implants. • Compatible with MRI and CT imaging devices. • Can reversibly modulate brain activities.	• Parameters for excitation versus inhibition neuromodulation require further research. • Potential cellular, molecular, synaptic, and ionic mechanisms of LIFU neuromodulation should be investigated.

*Penetration depth depends on nanoparticle placement.

LIFU emerges as a leading neuromodulation technique due to its precision, safety, and versatility. It boasts millimeter precision, facilitating targeted modulation of specific neural populations within the brain and minimizing unintended impacts on surrounding tissues (91, 92). LIFU’s mechanism of action involves direct interaction with neuronal tissue through mechanical energy, which allows deeper tissue penetration than techniques using electrical and magnetic field interactions (93, 94). Thus, LIFU can access deeper brain structures, including subcortical nuclei, making it valuable for addressing conditions like SUD (95, 96).

LIFU has a good safety profile when operated within minimal and nonsignificant risk recommendations of the International Transcranial Ultrasonic Stimulation Safety and Standards Consortium (ITRUSST) (97). LIFU operates at frequencies that prevent heat generation, mitigating the risk of magnetothermal lesions associated with high-intensity magnetic fields used in specific TMS and NEMS protocols (20, 98). LIFU also reduces potential complications like immune responses to nanoparticles, which may arise in NEMS technology (98). This favorable safety profile makes it a more biocompatible and patient-friendly neuromodulation technique. While NIR optogenetic stimulation offers high spatial resolution, its application requires invasive genetic modification of targeted cells, limiting its versatility (99). In contrast, LIFU presents a noninvasive approach that does not require genetic manipulation and nanoparticle injection, simplifying its application and broadening its suitability across a diverse spectrum of neurological disorders.

One of the unique and critical advantages of LIFU is its capability for bidirectional modulation of neural activity. LIFU can reversibly inhibit or excite neuronal circuits depending on the parameters applied (92). The flexibility offered by LIFU is particularly relevant in treating SUD. In addition, specific circuits may be hyperactive (during late withdrawal) or underactive (during early withdrawal). Adjusting LIFU’s parameters makes it possible to tailor the treatment to the individual’s neurophysiological SUD profile.

### 3.2 Molecular action mechanism of LIFU neuromodulation

Neurons are viscoelastic materials capable of propagating mechanical energy and storing it elastically (100). Mechanical interactions have led to the proposal of several mechanisms of action for LIFU, including mechanosensation, electrophysiological-mechanical coupling, microtubule resonance, thermal mechanism, and cavitation.

Mechanosensation converts mechanical energy into neural signals through mechanosensitive ion channels. LIFU is hypothesized to physically displace and activate these channels, leading to changes in ion transport, neuronal depolarization, and altered neural signaling (101–103). LIFU has been shown to interact with calcium-permeable mechanosensitive channels like TRPP1/2, TRPC1, TRPA1, and Piezo1 (101–104). Additionally, mechanosensitive ion channels from the two-pore-domain potassium channel family (e.g., TREK-1 and TRAAK) have been shown to respond to LIFU (100, 105). LIFU can also activate touch sensation MEC-4 channels (including DEG/ENaC/ASIC ion channel), large conductance mechanosensitive channel MscL, and sodium ion channels (106). Cytoskeleton also plays a part in the LIFU mechanosensation mechanism. Duque et al. recorded calcium influx and membrane currents in hsTRPA1-expressing cells of rats and mice, likely due to the interaction of the sonication-sensitive N-terminal tip domain of hsTRPA1 with the actin cytoskeleton (107). In another study, acoustic pressure waves were generated when LIFU traveled through the extracellular matrix, ultimately activating ASIC1a in a cytoskeleton-dependent manner (102). This activation likely occurs in concert with the simultaneous activation of other mechanoreceptors, suggesting a complex interplay within the mechanosensitive system underlying LIFU’s actions. However, only a few mechanosensitive ion channels have been studied for LIFU’s effects.

Beyond individual channels, LIFU modulates broader mechanosensitive machinery, leading to changes in mechanically coupled electrophysiological signals. These signals are associated with changes in membrane conformational state changes and mechanosensitive ion channels. Changes in membrane conformational states involve mechanical signals influenced by surface tension, elasticity, and intracellular fluid viscosity (108). These conformational changes can be externally induced via LIFU’s mechanical energy, altering membrane fluidity and permeability (109). This high-energy state causes embedded proteins and lipids to adapt, changing the membrane’s capacitance and modulating neural activity. Mechanical deformations redistribute dipoles in lipid bilayers in neuronal membranes, causing surface polarization- a process termed direct flexoelectricity (DF) (110). LIFU’s mechanical energy provides a possible membrane deformation, which could lead to DF. Exogenous LIFU pulsation could also interfere with these native thermodynamic waves generated by lipid phase transition by transferring acoustic energy and generating pressure waves, which could alter action potentials depending on the neuron’s initial state and orientation (111, 112).

LIFU stimulation can also cause cavitation, where gas bubbles within tissues resonate, expand, and collapse depending on the frequency, creating mechanical effects. Though bubbles are generally negligible in the nervous system, micro cavitation can increase membrane permeability via sonoporation, creating pores in the lipid bilayer. At the same time, mechanosensitive channels could be activated during micro cavitation. LIFU combined with ultrasound microbubble contrast agents could have controllable cavitation effects. However, clear models for this application are still being developed. The neuronal intramembrane cavitation excitation (NICE) models predicts cell-type-specific responses that correlate indirectly with experimental data, and the SONIC model addressed the computational speed limitation of the NICE model (113–115). Both models describe how US-induced cavitation can modulate neuronal activity. However, many studies obtain results that do not follow the predictions of these models partly because LIFU stimulation does not always target singular cell types at a time.

So far, the effects of LIFU have mostly been routed through mechanical interactions. In addition to the earlier described mechanisms, Hameroff et al. propose that the LIFU in specific megahertz frequency bands can resonate with microtubules, causing them to vibrate when aligned with their long axis (116). Even with these microtubule vibrations, electrophysiological-mechanical coupling, and cavitation are likely. Some studies have proposed thermal mechanisms. For example, Darrow et al. suggest a 2°C increase might contribute to neuro inhibition under specific conditions (117). However, unlike HIFU, which utilizes high intensities for tissue ablation, direct LIFU stimulations typically induce negligible temperature increases (<1°C), which is generally considered insufficient for direct neuronal modulation (117, 118). Therefore, thermal effects are unlikely to be the primary driver, but thermal modeling and reported thermal indices remain valuable in accounting for variations in sonication parameters, tissue properties, and beam configurations during treatment optimization. While LIFU has been used for various successful applications of neuromodulation, much work remains to be done to understand the complex interactions that account for LIFU’s action mechanisms with varying parameters.

## 4 Neuromodulatory effects of LIFU parameters on addiction-related brain regions

Optimizing LIFU for SUD treatment demands a comprehensive grasp of how diverse parameters influence its neuromodulatory effects within specific brain regions. These parameters determine whether LIFU yields inhibitory or excitatory responses, fundamentally shaping treatment efficacy. However, challenges arise due to discrepancies in parameter reporting, complicating efforts to replicate and compare parameters. To this end, the ITRUSST suggested a guideline for standardized reporting of ultrasound parameters (119). We have based the discussion in this section on the proposed guidelines. **Table 2** presents definitions, abbreviations, and units for each parameter set to facilitate clarity and standardization.

**Table 2:** Definitions of ultrasound parameters.

Parameter Type	Parameter Name	Definition	Abbreviation	Unit
Frequency	Transducer center frequency	Frequency at transducer focal center	*f*_c_	Hz
	Operating Frequency	Driving frequency of the transducer	*f*_0_	
	Pulse repetition frequency	The inverse of pulse repetition interval	PRF	Hz
	Pulse train repetition frequency	The inverse of Pulse train repitition interval	PRF_pulse train_	Hz
Intensity	Spatial-peak pressure amplitude	Peak acoustic pressure	*p*	Pa
	Spatial-peak, pulse-averaged	time-averaged intensity over the pulse duration	I_sppa_	W/cm^2^
	Spatial-peak, time-averaged intensity	Product of DC_pulse train_ and I_sppa_	I_spta_	mW/cm^2^
Duration	Pulse duration	Total on time for individual pulse	PD	s
	Pulse Repetition Interval	The time between two pulses in a pulse train	PRI	s
	Pulse Train Duration	Total time for pulse train	PTD	s
	Pulse Train Repetition Interval	The time between each pulse train	PTRI	s
	Total Sonication Duration	Total time for all pulse trains	TSD	s
Duty Cycle	Pulse Train Duty Cycle	Percentage ratio of PD and PRI	DC_pulse train_	%
	Pulse Train Repeat Duty Cycle	Percentage ratio of PTD and PTRI	DC_pulse train repeat_	%
	Duty Cycle	Product of DC_pulse train_ and DC_pulse train repeat_	DC	%
Others	Mechanical index	The ratio of peak-refractional pressure and the square root of *f*_0_	MI	–
	Thermal index	The ratio of the power used to that required to raise tissue temperature by 1°C	TI	–

### 4.1 Frequency

**Figure 1:**
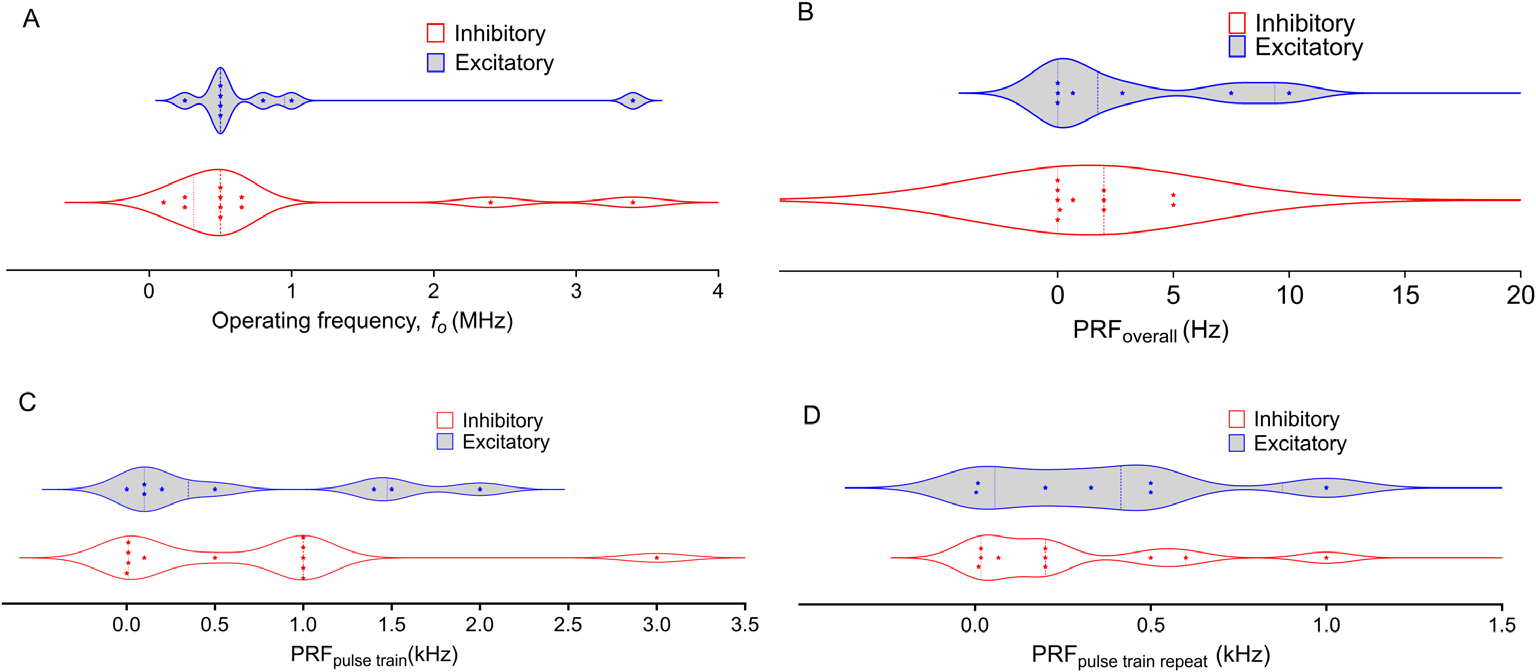
Distribution of Operating Frequency and Pulse Repetition Frequency (PRF) in Low-Intensity Focused Ultrasound (LIFU) treatments across NAc, PFC, AMY, and HP targets. The plots differentiate between inhibitory (blue) and excitatory (red) protocols, with each data point representing a distinct study. Dashed lines represent the first and third quartiles, with the middle-dashed line indicating the median. **(A)** Operating Frequency varies widely, with inhibitory frequencies concentrated at lower values and greater variability in excitatory frequencies. **(B)**. Most Overall PRFs are below 5 Hz, except for two excitatory studies reporting higher values, showing a trend toward lower PRFs across LIFU protocols. **(C)**: Pulse Train PRF exhibits some variability, but both inhibitory and excitatory protocols tend to cluster around lower frequencies. **(D)**: Pulse Train Repeat PRF shows a broader distribution, with a more even spread between inhibitory and excitatory protocols compared to other panels. The violin plots highlight the spread and density of the data, illustrating the diversity of frequency parameters used in LIFU treatments across different brain regions and experimental conditions.

Ultrasound is applied using transducers with specific fundamental frequencies or acoustic frequencies to target brain regions, and neuromodulation with LIFU relies on specific frequencies for precise targeting of brain regions. While LIFU studies have used *f*_0_ ranging from 200 kHz to 10 MHz (**Figure 1**), compared to higher *f*_0_ for medical imaging (around 15 MHz), skull properties present a significant challenge. The acoustic pressure weakens through the skull due to the conversion of sound waves, absorption by bone, reflection, and scattering. Standing waves can also form on the other side of the skull, potentially causing unintended sonication effects. Acoustic pressure attenuation is particularly problematic when using higher frequencies, which offer excellent spatial resolution but penetrate less deeply. In contrast, while a low fundamental frequency is favorable for deeper targets, excessively low values make ultrasound waves travel deeper than intended, potentially reaching unintended brain regions. Therefore, the fundamental frequency is in the middle range of frequencies to allow sufficient penetration to reach the target region, and avoiding these complications is vital to successful LIFU applications.

The pulse repetition frequency (PRF) is the rate at which ultrasound pulses are delivered. According to ITRUSST, the PRF could be reported as either the individual pulses in a pulse train (PRF_pulse train_) or the frequency at which the pulse train is repeated (PRF_pulse train repeat_) (119). Both PRF_pulse train_ and PRF_pulse train repeat_ are crucial parameters in focused ultrasound, as they define the pattern of every ultrasound waveform. While PRF_pulse train_ influences the observed effect of LIFU, PRF_pulse train repeat_ is usually employed to avoid excessive heat buildup. However, repeated stimuli separated by fixed times can initiate Long-Term Potentiation (LTP) and long-term memory (LTM) encoding (120, 121). For instance, we have previously used a 0.2 Hz PRF_pulse train repeat_ in the downstream inhibition of dopamine to avoid heating with a 10 MHz *f*_c_ transducer (122). A theta burst protocol was also developed by Zeng et al. to induce consistent corticospinal excitability (123).

While the exact influences of PRFs are still under investigation, a growing body of research suggests that PRF is more than an on/off switch for inhibition or excitation. Stimulation with 250 kHz and 1 kHz PRF_pulse train_ can suppress NAc activity, suggesting a wide range of possible inhibitory PRF_pulse train_ (124, 125). In recent studies, the He group used 3 kHz to depress field excitatory postsynaptic potentials in rats HP, Kim et al. utilized a 3 kHz PRF_pulse train_ to reduce heat pain sensitivity by targeting human IC, and the Legon group achieved similar effects using a 1 kHz PRF_pulse train_ (126–129). However, Niu et al. showed excitation of GluA1 expression alongside inhibition of GluA2 and GluA3 in mice NAc with 1 kHz (130). Therefore, predicting inhibitory vs excitatory mechanisms is challenging.

In the PFC, the complexity increases. Our group found that applying a 1 kHz PRF_pulse train_ to the PFC led to downstream dopamine inhibition in the NAc, and 1.5 kHz targeting the PFC has been used to inhibit inflammation in rodents (122, 131). PRF_pulse train_ lower than 1 kHz have also been used in neuromodulation. Yi et al. showed downregulation of inflammation markers with 100 PRF_pulse train_ (132). Huang et al. showed excitation of postsynaptic current and GluN2A expression with 500 Hz, Ren et al. used 200 Hz to reverse the expression of depression-related genes in the PFC, and the Lee group showed excitation at 140 Hz and inhibition at 10 Hz PRF_pulse train_ (133–135). Similarly, Pan et al. used a PRF_pulse train_ of 1 Hz to increase neuronal activity in mPFC cells, and Xie et al. showed an increased firing rate upon LIFU sonication at the same PRF_pulse train_ (136, 137). Chou et al. and Kuhn et al. used a 10 Hz PRF to inhibit functional connectivity in the amygdala (138, 139). However, a PRF_pulse train_ of 1 kHz produced a similar result in a study by Folloni et al. (140) From the distributions depicted in **Figure 1**, the PRF_pulse train_ from both excitation and inhibition span similar ranges. The same trend is observed for the PRF_pulse train repeat_. These findings suggest that PRF is not the sole determinant of inhibition or excitation in LIFU applications.

It is crucial to consider that each brain region in every organism has a natural local oscillation frequency (141). However, it is noteworthy that apart from the studies by Chou et al. and Kuhn et al., which used 10 Hz, and Xie et al., and Pan et al., which used 1 Hz, the PRFs tested to date predominantly fall within the high gamma domain or high-frequency oscillation domains (128, 133, 137). This observation raises the question of how LIFU might interact with other frequency domains, such as delta, theta, alpha, and beta, each known to correlate with specific tasks (142, 143). EEG spectral measurements indicate that diseases and behavioral changes may shift native oscillation frequencies (144–146). Consequently, the susceptibility of different brain areas to specific PRF ranges could be dictated by the constructive or destructive interference of LIFU waves with the specific brain waves of a particular region (141). This complex interplay of factors underscores the need for further research to understand the full implications of PRF in LIFU applications. Future research should delve deeper into the interplay between pulse repetition frequency (PRF), other parameters, and brain region, to understand its neuromodulatory effects to unlock the full potential of LIFU therapy.

### 4.2 Duration and duty cycle

There are three different layers of duration and duty cycle in the LIFU parameter selection. The pulse/pulse train/total sonication duration (PD/PTD/TSD) refers to the time-on duration of the pulse/pulse train/sonication waveform. At the same time, the duty cycle is the percentage ratio between pulse/burst/sonication duration and the total pulse/burst/sonication period. Notably, while some studies employ both pulse train and pulse duration in parameter selection, others use either pulse or burst duration. There is a wide range of PD and PTD, from microseconds (5 µs) to milliseconds (360 ms). For instance, Mahoney et al. employed 100/900 ms (on/off) PD in humans, and Niu et al. used 5 μs pulse duration in mice (130, 147). There is a broader range of pulse train duration values (1 ms - 5 min). TSD also varies widely (40 s- 1 hour). Mahoney et al. applied LIFU for 10 minutes per hemisphere in humans, whereas Deveci et al. used a longer 30-minute duration in rats (124, 147). In the studies highlighted in **Table 3**, excitatory or inhibitory effects are not necessarily favored by shorter or longer TSD. Kim et al. observed both excitation and inhibition with 20 min TSD, while Lin et al. found that 20 min of LIFU could lead to excitation in neuronal activity and reduction in drug-seeking (134, 148). However, the choice of TSD is crucial as it appears to determine the level of effects observed. For example, Deveci et al. observed reduced alcohol SUD-related gene expression from 454 gene expression changes after short-term stimulation to 382 gene expression changes after long-term stimulation (124). There may be a correlation between the effectiveness of long-term stimulation and the process of encoding long-term memories. This process usually occurs in a time scale of minutes (120, 121).

**Table 3:** LIFU studies on common drug addiction-related brain regions.

Article	Relevant Brain Region	Frequency	Duration	Intensity	Sonication Effect
Mahoney et al.	NAc (Humans)	*f*_0_: PRF: 220 kHz PTRF:0.067 Hz, 0.033 Hz	PD:100 ms PRI:1 sec PTD: 5 sec, 10 sec PTRI: 15 sec, 30 sec TSD: 10 min DC_pulse train_: 10% DC_pulse train repeat_: 3.3%	80 W/cm^2^ 55 W/cm^2^	• LIFU reduced cravings for alcohol, psychostimulants, and opioids for up to 90 days post-LIFU sonication.
Deveci et al.	NAc (Rats)	*f*_0_: 2.4 MHz PRF: 250 kHz PTRF: 0.6Hz	PD: 2 μs PRI: 4 μs PTD: 300 ms PTRI: 1700 ms TSD:30 min DC_pulse train_: 50% DC_pulse train repeat_: 17.6%	4.06 W/cm^2^ I_sppa_ 305m W/cm^2^ I_spta_	• Inhibition of alcohol dependency-related genes
Lan et al.	NAc (Mice)	*f*_0_: 0.5 MHz PRF: 1 kHz PTRF: 0.33 Hz	PD: 0.5 ms PRI: 1 ms PTD: 300 ms PTRI: 3 sec TSD: 10 min DC_pulse train_:50% DC_pulse train repeat_: 10%	590 kPa	• Inhibition of monoamine neurotransmitters • Cell damage potentially due to high acoustic pressure
Niu et al.	NAc (Mice)	*f*_0_: 3.4 MHz PRF: 0.1 kHz, 1 kHz PTRF: 0.5 Hz	PD: 50 μs PRI: 1 ms PTD: 1 s PTRI: 2 s DC_pulse train_:5% DC_pulse train repeat_: 50%	304 kPa	• GluA1 excitation • GluA2 and A3 inhibition. • Morphine-induced place preference suppression.
Lin et al.	IL (Rats)	*f*_0_ 0.5 MHz PRF: 500 Hz PTRF: 1 Hz	PRI: 2 ms PTD: 2 ms PTRI: 1 s TSD: 20 min DC_pulse train_:5% DC_pulse train repeat_: 50%	328 ​kPa	• Reduction in methamphetamine-induced place preference suppression • Excitation of cFOS expression
Olaitan et al.	PL (Rats)	*f*_0_: 10 MHz PRF: 1kHz PTRF: 0.2 Hz	PD: 360 ms PRI: 1 sec PTD: 500 ms PTRI: 5 sec TSD: 2 mins DC_pulse train_:36% DC_pulse train repeat_: 10%	13 W/cm^2^ I_sppa_	• Downstream dopamine Inhibition
Pan et al.	PFC (Rats)	*f*_0_: 1-MHz PRF: 1 Hz PTRF: 3.3 mHz	PD: 50 ms PRI: 1 s PTD: 50 ms PTRI: 5 mins TSD: 15 mins DC: 5% DC_pulse train_:5% DC_pulse train repeat_: 10%	528 mw/cm^2^ I_spta_	• improved anxiety-like behavior • Upregulated NMDA receptor and cFOS.
Kim et al.	mPFC (Humans)	*f*_0_: 250kHz PRF: 1.4 kHz, 100 Hz PTRF: 0.2 Hz	PD:0.5 ms PRI: 0.71 ms, 10 ms PTD: 300 ms PTRI: 5 sec, 0 sec TSD: 20 mins DC_pulse train_: 70%, 5% DC_pulse train repeat_: 6%, 100%	3 W/cm^2^	• Beta band power increased with excitatory stimulation (70% DC) • Theta band power increased with suppressive stimulation (5% DC)
Y. Wang et al.	mPFC (Mice)	*f*_0_: 0.5 MHz PRF:1.5 kHz PTRF: 0.5 Hz	PD: 0.1 ms PRI: 0.67 ms PTD: 0.5 s PTRI: 2 s TSD:15 mins DC_pulse train_:14.9% DC_pulse train repeat_: 25%	20 kPa	• Inhibited social avoidance • Enhanced mPFC neuronal excitation • Inhibited Microglial cell activation • Inhibited inflammatory pathway
Yi et al.	(Mice) PFC	*f*_0_: 0.5 MHz PRF:100 Hz PTRF: 8.3 mHz	PD: 5 ms PRI: 10 ms PTD: 60 s PTRI: 120 s TSD: 30 min DC_pulse train_: 50% DC_pulse train repeat_: 50%%	10.09 W/cm^2^ I_spta_	• Alleviates Depression and Anxiety-like behaviors • Downregulated Inflation Markers
Ren et al.	PFC (Rats)	*f*_0_: 800 kHz PRF:200 Hz PTRF: 0.33 Hz	PD: 0.2 ms PRI: 5 ms PTD: 1 sec PTRI: 3 sec TSD:20 min DC_pulse train_: 4% DC_pulse train repeat_: 33%	3.84 W/cm^2^ I_sppa_ 154 mW/cm^2^ I_spta_	• Reverses depression-related gene expression
Wang et al.	mPFC	*f*_0_: 0.5 MHz PRF:2 kHz PTRF: 0.5 Hz	PD: 0.3 ms PRI: 0.5 ms PTD: 0.5 sec PTRI: 2 sec TSD: 15 min DC_pulse train_: 60% DC_pulse train repeat_: 25%	500 and 230 mW/cm^2^ I_spta_	• Improved depression-like behaviors in CUS rats • Ameliorates the synaptic structural plasticity in the mPFC of CUS rats • Increased expression of mPFC post-synaptic proteins
Chou et al.	AMY Insula (Humans)	*f*_0_: 0.65 MHz PRF: 10 Hz PTRF: 16.7 mHz	PD: 5 ms PRI: 100 ms PTD: 30 sec PTRI: 60 sec TSD:20 min DC_pulse train_: 5% DC_pulse train repeat_: 50%	14.4 W/cm^2^ I_sppa_ 0.72 W/cm^2^ I_spta_	• Decreased blood-oxygen-level-dependent fMRI activation during fear task • decreased AMY-IC functional connectivity
Kuhn et al.	AMY	*f*_0_: 0.65 MHz PRF: 10 Hz PTRF: 16.7 mHz	PD: 5 ms PRI: 100 ms PTD: 30 sec PTRI: 60 s TSD: 5 min DC_pulse train_: 5% DC_pulse train repeat_: 50%	720 mW/cm^2^ I_spta_	• AMY-focused LIFU increased AMY and PFC perfusion • AMY-focused LIFU decreased functional right AMY-mPFC connectivity
Folloni et al.	AMY (Macaque)	*f*_0_: 250 kHz PRF: 10 Hz	PD: 30 ms PRI: 100 ms TSD: 40 s DC_pulse train_: 30% DC_pulse train repeat_: 50%	64.9 W/cm^2^ I_sppa_, 19.5 W/cm^2^ I_spta_ 1.44 MPa	• AMY functional connectivity is reduced after LIFU.
Zhu et al.	AMY	*f*_0_: 0.5 MHz PRF:1 kHz PTRF: 0.2 Hz	PD: 500-µs PRI: 1 ms PTD: 50/250/500 ms PTRI: 5 sec DC_pulse train_: 50% DC_pulse train repeat_:	0.2/0.4/0.8 mpa	• • Piezo1 knockout significantly reduced LIFU-induced neuronal calcium response.
Lim et al.	AMY *PFC* HP	*f*_0_: 1 MHz PRF:1 kHz PTRF: 66.67 mHz	PD: 500-µs PRI: 1 ms PTD: 300 ms PTRI:15 sec DC: 0.05%, 100%	7.4 mW/cm^2^ I_sppa_ 5 mW/cm^2^ I_spta_	• LIFU-mediated mechanotransduction and neuron activation were inhibited by ASIC1a blockade and cytoskeleton-modified agents.
In et al.	IC (Humans)	*f*_0_: 500 kHz PRF: 1 kHz PTRF: 0.2 Hz	PD: 360 ms PRI: 1 s PTD: 1 sec PTRI: 5 sec TSD: 10 mins DC_pulse train_: 36% DC_pulse train repeat_: 20%	4.5 W/cm^2^ I_sppa_ 1.5 mW/cm^2^ I_spta_	• LIFU to the PIC significantly attenuated pain ratings. • LIFU to AIC did not affect either TSP or CPM pain ratings.
Kim et al.	IC	*f*_0_: 1.5 MHz PRF: 40 Hz, 3 kHz PTRF: 0.5 Hz, 0.25 Hz	PD: 200 µs PRI: 25 ms, 0.33 ms PTD: 100 ms, 400 ms PTRI: 2 sec, 4 sec TSD: 10 mins, 20 mins, 1 hour DC_pulse train_: 0.8%, 60% DC_pulse train repeat_: 5%, 10%	208 mW/cm^2^ I_spta_ 0.35 W/cm^2^ I_sppa_	• Sustained behavioral change associated with heat hypersensitivity by targeting insula
Legon et al.	IC (Humans)	*f*_0_: 500 kHz PRF: 1 kHz	PD: 360 ms PRI: 1 sec PTD: DC: 36%	3.5 W/cm^2^	• LIFU to both AIC and PIC reduced perceived pain ratings • PIC responded 200 ms faster to LIFU than AIC • Delta, Theta, and Alpha EEG power bands are reduced in the AI
Xie et al.	HP (Rats)	*f*_0_: 0.5 MHz PRF: 1 Hz	PD: 50 ms PRI: 1 sec TSD: 15 min DC: 5%	8.66 W/cm^2^ I_sppa_ 0.43 W/cm^2^ I_spta_	• Improved the memory performance in the Y-maze behavior experiment. • Increased neuronal firing rate
Niu et al.	HP (Rats)	*f*_0_: 500 kHz PRF: 3 kHz PTRF: 50 Hz	PD: 167 μs PTD: 10 PTRI: 20 ms TSD: 5 min DC_pulse train_: 5% DC_pulse train repeat_: 50%	425 mW/cm^2^ I_sppa_ 255 mW/cm^2^ I_spta_ 99 kPa	• Depression of field excitatory postsynaptic potentials
Huang et al.	HP (Rats)	*f*_0_: 0.5 MHz PRF: 500 Hz	PD: 0.1 ms PRI: 2 ms TSD: 10 min DC_pulse train_: 5%	7.2 W/cm^2^ I_sppa_ 360 mW/cm^2^ I_spta_ 0.43 MPa	• Increased the density of dendritic spines. • Increased frequency of spontaneous excitatory postsynaptic current. • Increase in the expression level of GluN2A.

**Figure 2:**
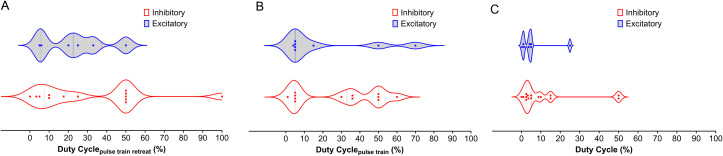
Distribution of Duty Cycle Across NAc, PFC, AMY, and HP LIFU Treatments. This figure depicts the distribution of duty cycle for inhibitory and excitatory frequencies across different brain regions (NAc, PFC, AMY, and HP) treated with LIFU. **(A)** Duty cycle for the pulse train retreat. There is a significant overlap between inhibitory and excitatory duty cycles for the pulse train retreat, with no clear trend. **(B)** Duty cycle for the pulse train. Inhibitory duty cycles for the pulse train tend to be higher than excitatory duty cycles. **(C)** Duty cycle for the entire pulse train. Duty cycles for the entire pulse train show a similar trend to Panel B, with inhibitory duty cycles generally higher than excitatory duty cycles. Each data point represents a separate study. The violin plots illustrate the variability in duty cycle parameters, with the median represented by the solid line and the quartiles indicated by the dashed lines.

The duty cycle is a function of both duration and repetition frequency (**Table 2**). As with other parameters, the duty cycle may have been chosen based on the targeted brain region, desired effects (excitation vs. inhibition), and safety considerations. Studies on humans focus on safety and tolerability, while studies on animals have more flexibility in exploring different stimulation parameters, including the duty cycle. The parameters might involve continuous or near-continuous LIFU for initial assessment, and pulsed LIFU with varying duty cycles. Lower duty cycles have been thought to cause inhibitory effects, while higher duty cycles lead to excitatory effects (92). A similar trend is observed in the studies by Kim et al. and Wang et al., where DC_pulse train_ of 70 and 60% cause excitation (134, 149). However, Kim et al. also observed excitatory effects with a DC_pulse train_ of 0.8% and a DC_pulse train repeat_ of 10%, an exception to this trend. Moreover, **Figure 2** shows that more studies found inhibition at DC_overall_ greater than 10% compared to excitation. A similar trend is observed with the DC_pulse train repeat_. Therefore, there is no conclusive rule for duty cycle selection. As with the PRF, each brain region could possess a natural duty cycle for wave oscillations, and selected LIFU parameters could lead to constructive or destructive interference, leading to the neuromodulatory effect. However, further research is needed to establish these relationships and optimize LIFU protocols for targeted modulation of neural circuits in SUD.

### 4.3 Intensity

Intensity is a critical parameter influencing LIFU neuromodulatory effects. It is typically quantified in units of either Isppa (spatial peak intensity averaged) or Ispta (spatial peak temporal averaged). While some studies report acoustic pressure instead of intensity, and I_sppa_ and I_spta_ are not always differentiated, they convey the same concept. Studies investigating direct NAc stimulation with 4.06 W/cm^2^ I_sppa_ [0.305 W/cm^2^ I_spta_] and acoustic pressures of 590 kPa have demonstrated suppression of NAc gene expression and activity (124, 125, 130). Conversely, 304 KPa targeted at the NAc showed simultaneous inhibition and excitation of different subunits of AMPA receptors, ultimately allowing ion flow.

The effects on the PFC are more nuanced. Different PFC regions stimulated with LIFU can exhibit direct and downstream excitatory or inhibitory effects. Our previous work demonstrated effective downstream inhibition of NAc dopamine release via PFC sonication at 13 W/cm^2^ (122). Wang et al. reported inhibitory effects of LIFU with at 500 mW/cm^2^, Pan et al. reported upregulation of the NR1 NMDA receptor and cFOS downregulation in the mPFC at 500 mW/cm^2^, while Kim et al. observed both inhibitory and excitatory effects on the PFC at 3 W/cm^2^ (131, 134, 136). Notably, several studies have achieved excitatory effects on the PFC using various intensities (3 W/cm^2^ I_sppa_, 20 kPa, and 328 kPa) (131, 134, 148). For the insular cortex, stimulation with intensities of 3.5 W/cm^2^, 208.46 mW/cm^2^, and 300 kPa resulted in inhibition, while 8.66 and 0.35 W/cm^2^ yielded excitation (126–128). Additionally, Chou et al. employed 14.4 W/cm^2^ I_sppa_ (0.72 W/cm^2^ I_spta_) to decrease Amygdala-IC functional connectivity, while the Legon group used 3.5 W/cm^2^ I_sppa_ to attenuate pain ratings in the insula (127, 138).

**Figure 3:**
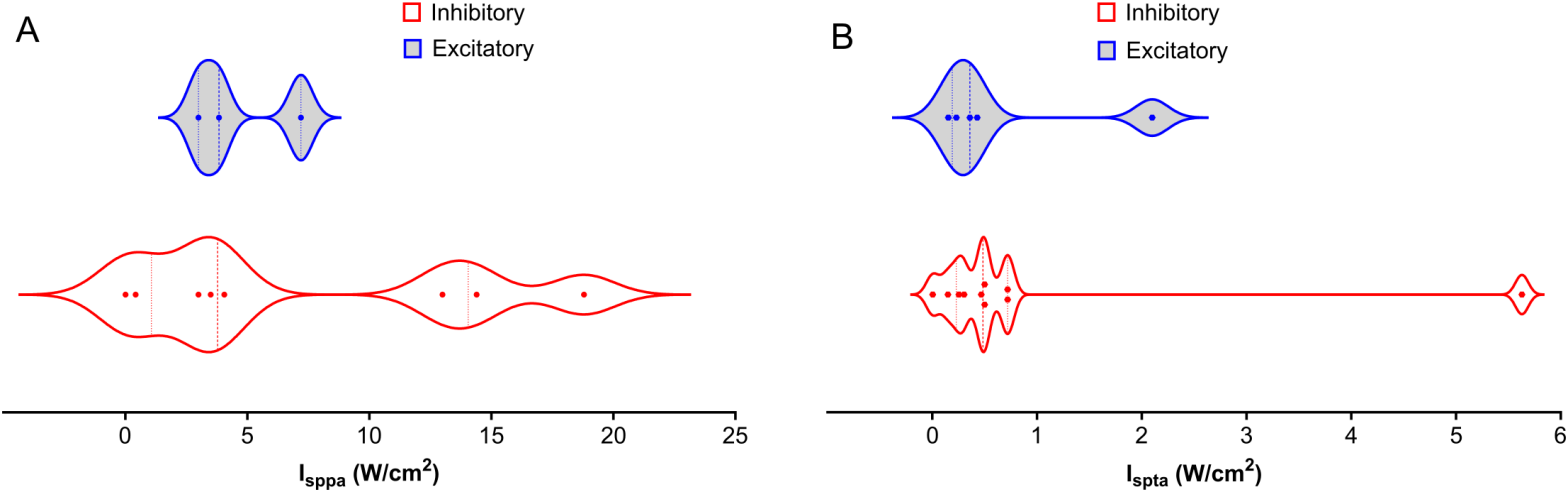
Distribution of Intensities Across NAc, PFC, AMY, and HP LIFU Treatments. This figure depicts the distribution of spatial peak intensity (Isppa) and spatial average intensity (I_spta_) for inhibitory and excitatory frequencies across different brain regions (NAc, PFC, AMY, and HP) treated with LIFU. **(A)** Isppa (W/cm^2^) distribution. There is a significant overlap between inhibitory and excitatory Isppa values, with no clear trend **(B)** I_spta_ (W/cm^2^) distribution. I_spta_ values for inhibitory treatments tend to be higher than those for excitatory treatments. Each data point represents a separate study. The violin plots illustrate the variability in intensity parameters, with the median represented by the solid line and the quartiles indicated by the dashed lines.

Many specific studies find effects of intensity, but trends across studies are difficult to elucidate. **Figure 3** shows a wide range of distribution of I_sppa_ variation between 0- 90 W/cm^2^ across studies. I_spta_ applied is generally lower than 1 W/cm^2^ partly due to FDA regulations. Consequently, safety becomes the primary concern regarding LIFU intensity for SUD treatment. Higher intensities are generally associated with higher tissue heating, but the exact relationship between intensity and therapeutic effect remains under investigation. Future research should prioritize establishing optimal intensity thresholds within brain regions relevant to SUD.

## 5 LIFU as an intervention for drug addiction treatment

LIFU stands at the vanguard of neuromodulatory interventions for SUD treatment, offering hope for reversing the debilitating neurological sequelae of SUDs (150). The efficacy of LIFU extends beyond mere symptom management; it delves into the core of SUD behavior, attenuating the relentless cravings that fuel the cycle of abuse. Research has illuminated LIFU’s capacity to modulate neural circuits, thereby diminishing drug-seeking behaviors in both animal models and human subjects (150). Moreover, LIFU’s influence has implications on the social and emotional behaviors of SUD patients, alleviating associated fear and anxiety, which are often comorbid with SUDs (131, 138). This neuromodulation may foster synaptic plasticity, potentially recalibrating the molecular mechanisms disrupted by prolonged substance abuse. The promise shown by LIFU in these preliminary studies paves the way for its consideration as a noninvasive adjunctive therapy, potentially reducing cravings, restoring functional outcomes, and improving the overall quality of life for individuals battling SUD.

### 5.1 Effect of LIFU on addictive behavior

**Figure 4:**
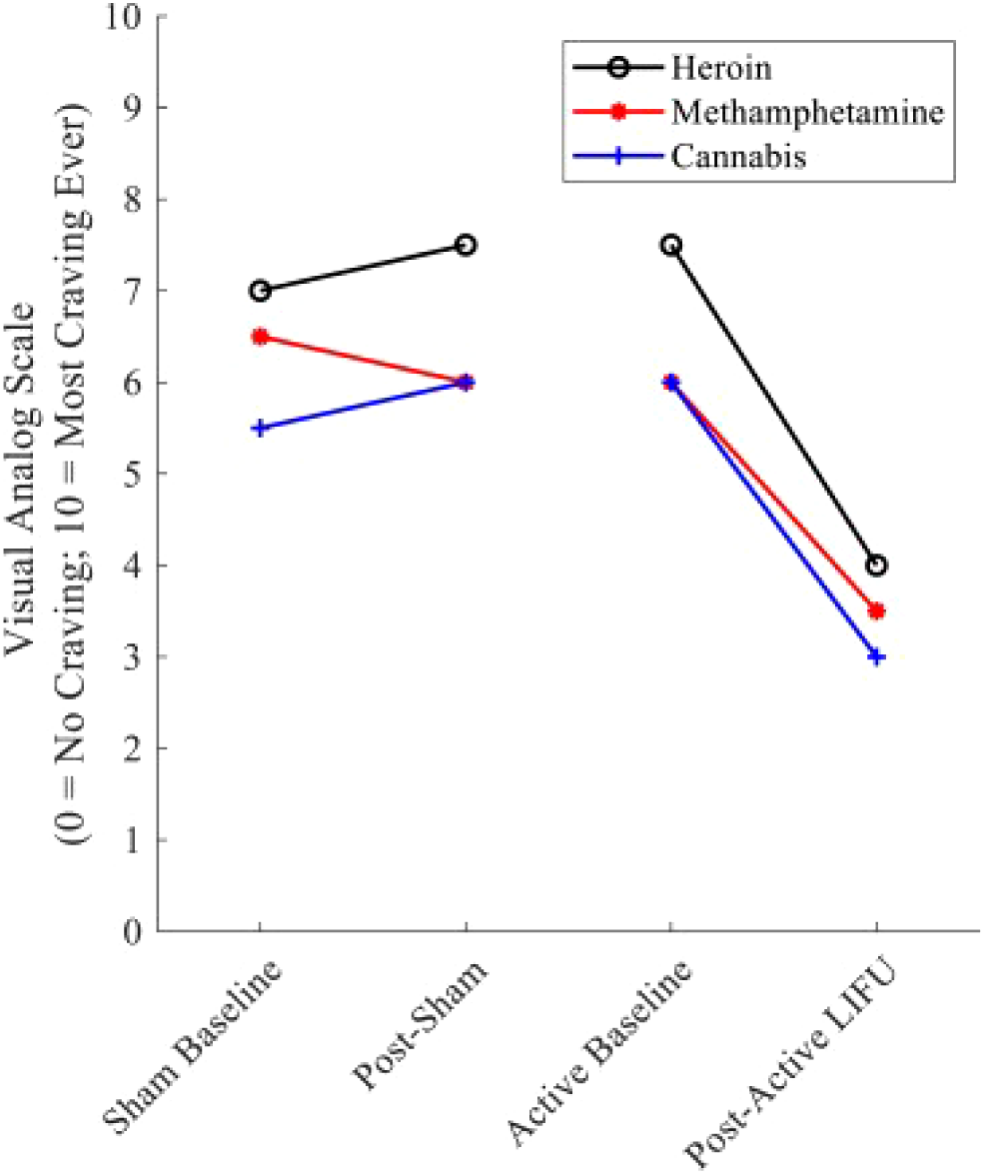
Substance craving rating pre—and post–LIFU: sham vs. active sonication. Values represent cue-induced craving ratings immediately before and after sham and active LIFU. Following sham LIFU sonication, minimal changes were noted in craving ratings. Following 10 minutes of active LIFU sonication, ∼craving for the participant’s primary substances of use was reduced by 50%. Craving Reduction was sustained for 90 days post-LIFU Sonication. Reprinted with permission from "Low-intensity focused ultrasound targeting the bilateral nucleus accumbens as a potential treatment for substance use disorder: A first-in-human report" by Mahoney, J. J. et al., (2023) licensed under © 2023 Society of Biological Psychiatry. (151).

Exploring the frontiers of SUD treatment, LIFU emerges as a promising tool, demonstrating significant potential in mitigating addictive behaviors as evidenced by recent scientific studies. LIFU’s reduction of craving has been shown in both animals and humans. Lin et al. highlighted LIFU’s potential in reducing methamphetamine (MA)-seeking behaviors in rats in a conditioned place preference (CPP) test model (148). Notably, LIFU applied to the infralimbic cortex was most effective in reducing MA-seeking behaviors induced by MA priming, with no observed impact on MA-induced locomotor activity. This observation suggests a specific effect of LIFU on craving behavior rather than general locomotion. Mahoney et al. explored the potential of LIFU neuromodulation for treating SUDs by targeting the bilateral NAc (**Figure 4**) (147, 151). A 43-year-old participant underwent both sham and active LIFU sonication, reporting an immediate reduction in cravings for primary substances. Cue-induced craving for alcohol and illicit substances was utterly suppressed during post-LIFU follow-up assessments up to 90 days, alongside the elimination of substance-related dreams and improvements in anxiety and overall functioning. Urine toxicology analyses confirmed abstinence throughout the follow-up. In a follow-up study, four participants with opioid use disorder underwent active LIFU targeting the NAc, showing reduced craving during and after treatment, with lasting reductions observed during a 90-day follow-up. These findings suggest that LIFU targeting the NAc is a safe and effective intervention for reducing substance craving and improving outcomes in SUDs. However, more extensive and randomized trials are needed to validate these findings and fully assess the impact of LIFU on substance use and relapse, potentially offering a noninvasive adjunctive therapy for SUDs with the potential to reduce cravings and improve treatment outcomes.

Addiction disrupts communication pathways between reward circuits and areas governing decision-making and impulse control, exacerbating the challenge of resisting cravings as reward circuits become hyperactive while decision-making areas become hypoactive. However, the brain’s inherent neuroplasticity presents both a hurdle and an opportunity in this context. While SUD rewires neural circuits, treatment interventions have the potential to restore them to a healthy state. Notably, studies indicate that LIFU modulates neuronal activity and influences responsiveness to other neural inputs, thereby facilitating observed alterations in activity coupling. For instance, Folloni et al. examined the impact of LIFU on neural activity in the human amygdala, revealing reduced activity coupling between the stimulated area and its interconnected regions (140). Wang et al. also showed improved theta oscillation synchronization after LIFU stimulation of the mPFC (149). Peng et al. used functional magnetic resonance imaging (fMRI) to investigate the effects of LIFU on the reward network in ten healthy adults (152). Bilateral NAc inhibition was observed during LIFU on the left NAc compared to sham, with increased functional connectivity between the NAc and medial PFC (mPFC). These findings suggest that LIFU is a promising tool for the direct and noninvasive modulation of the NAc, shedding new light on treating SUD and other brain diseases involving reward processing.

### 5.2 LIFU effects on introceptive signals

Many individuals with SUD initially turn to substances to cope with introceptive disorders, such as hypersensitivity to pain, social anxiety, fear, and depression. Addictive drugs can provide temporary relief from these interoceptive responses, leading to a cycle of self-medication and potential SUD. However, introceptive dysfunctions of the IC and ACC following chronic drug use amplify these responses. Therefore, even when such individuals withdraw from drug use, underlying emotional problems often lead to relapse (27). LIFU has exhibited promise in regulating anxiety, fear, and depression. In a study by Chou et al., Blood Oxygen Level-Dependent (BOLD) activation in several regions of the fear network, including the AMY, HP, and dorsal ACC, was altered by LIFU, suggesting the reorganization of functional connectivity of fear-related brain activity (138). Yi et al. found that LIFU ameliorates depressive-like behaviors and anxiety-like behaviors while inhibiting proinflammatory cytokine upregulation in the PFC (132). Similarly, Wang et al. observed amelioration of depression-like behaviors through improvements in theta oscillation synchronization and synaptic functional plasticity within the vCA1 - mPFC pathway following LIFU treatment (149). Wynn et al. demonstrated LIFU’s effectiveness in treating pain by applying LIFU to both anterior and posterior IC, attenuating contact heat evoked potentials (CHEP) peak-to-peak amplitudes, indicating a reduction in neural responses to the painful stimulus (127). Additionally, In et al. showed that LIFU to the PIC significantly attenuated pain ratings in both temporal summation of pain (TSP) and the conditioned pain modulation (CPM) protocols, while LIFU to AIC did not affect either TSP or CPM pain ratings (126). Wang et al. also demonstrated the alleviation of chronic allodynia pain by ACC inhibition, potentially offering a novel approach to managing pain hypersensitivity induced by opioid misuse (153).

### 5.3 LIFU’s effect on functional connectivity of neural pathways

LIFU holds the potential to recalibrate disrupted neural pathways and instigate molecular changes controlling synaptic plasticity. In a recent study by Kuhn et al., LIFU was used in conjunction with ASL MRI and simultaneous BOLD fMRI to investigate its impact on deep brain regions in humans (139). The results demonstrated that LIFU could selectively enhance regional blood flow and modulate network connectivity of subcortical regions in a targeted manner. Interestingly, tFUS parameters aimed at disrupting activity led to decreased functional connectivity in the amygdala network while increasing BOLD activity and functional connectivity in the targeted ErC and its network. Both LIFU protocols led to increased perfusion exclusively in the targeted brain region, highlighting the focal and region-specific modulatory effects of tFUS. Thus, LIFU selectively enhances regional perfusion while modulating regional activity and connectivity, opening up potential clinical applications for emotion regulation and memory function.

Substance use disorder, particularly involving fentanyl, can worsen neurodegenerative processes. Recent research by Lim et al. suggests that LIFU could promote neurogenesis in the brain, potentially aiding in treating neurodegeneration linked to SUD (102). However, additional research is needed to fully understand the extent to which LIFU can induce neurogenesis in the brain regions most impacted by SUD. Nonetheless, preclinical studies show that LIFU, a noninvasive neuromodulation technique, holds considerable promise for addressing SUD-related behavioral issues. While further research is necessary to determine its efficacy and safety in human populations, LIFU presents a promising new approach for developing novel therapeutic interventions.

## 6 Future directions for LIFU treatment of SUD

The preceding sections have highlighted the significant potential of LIFU in SUD treatment, specifically its ability to modulate brain regions linked to addictive behaviors. However, further investigation into the physiological mechanisms underlying LIFU’s effects is imperative to fully capitalize on its therapeutic benefits. A comprehensive understanding of how LIFU interacts with neural circuits and neurotransmitter systems implicated in substance use disorder (SUD) is necessary to optimize its therapeutic application. Furthermore, SUD results in diverse neurological sequelae influenced by various factors, including the type of drug, duration of use, and overall health status (28). Integrating LIFU with existing pharmacological, behavioral, and cognitive interventions has the potential for synergistic effects, enabling more holistic and personalized approaches to SUD management. Therefore, exploring the integration of LIFU with other SUD treatment modalities is essential to improving treatment outcomes.

### 6.1 Investigation of LIFU’s physiological mechanisms

The precise mechanisms underlying the modulation of neural circuits by LIFU warrant exploration, alongside elucidation of how such changes manifest in downstream behavioral effects. Prior investigations have focused on LIFU’s influence on calcium signaling and mechanosensitive ion channel expressions at the cellular and molecular levels (101, 102). However, LIFU modulation entails a complex interplay of neurophysiological processes beyond these aspects. Comprehensive mechanistic and behavioral studies are imperative to better understand the criteria for LIFU parameter selection. For example, studies should explore the effect of LIFU on upstream and downstream neurotransmission in SUD-related circuits. Methods to measure cell firing and real-time changes in neurotransmission are essential to comprehend the neurotransmission basis for observed behavioral effects of LIFU.

Moreover, mechanistic studies aimed at elucidating the underlying neural mechanisms of LIFU-induced neuromodulation in SUD-related behaviors should include the pre- and post-synaptic terminals, surrounding astrocytes, and presynaptic extracellular matrix between these elements. These studies may involve examining changes in neural activity, protein expression analysis, and real-time neurotransmitter changes following LIFU treatment. Understanding the precise molecular mechanisms and neural circuits involved in LIFU-mediated effects can inform targeted interventions and optimize treatment outcomes.

Once LIFU’s effect on neurotransmission and downstream molecular signaling cascades is established, further behavioral studies can assess the impact of LIFU treatment on SUD-related behaviors using preclinical animal models and clinical trials. Preclinical studies in rodents, mainly focusing on the NAc, have reported promising results, with LIFU application reducing drug-seeking behavior in models of cocaine, alcohol, and opioid dependence. However, further investigations on other abused substances, including fentanyl, are warranted. By targeting additional brain regions and investigating SUD in various addictive drugs, LIFU may offer comprehensive interventions for addressing multiple aspects of SUD pathology.

### 6.2 Integration of LIFU with other SUD treatment modalities

Utilizing low-energy sound waves, LIFU provides a noninvasive and targeted treatment modality when combined with microbubbles, tiny gas-filled spheres containing drugs that are introduced into the bloodstream. LIFU can be multiplexed with pharmacological treatments and could be used for targeted drug delivery for SUD and relapse treatments. This innovative approach may improve the delivery of drugs across the blood-brain barrier, a significant challenge in treating neurological conditions. By amplifying the effects of medications designed to alleviate SUD symptoms, such as cravings and withdrawal, LIFU holds promise as a potent therapeutic tool. Clinical trials, such as those conducted by InSightec, are currently exploring LIFU’s safety and efficacy as an adjunctive treatment for Opioid Use Disorder (OUD), potentially paving the way for broader applications in SUD treatment (154, 155).

Integrating LIFU with cognitive-behavioral therapy (CBT) and other behavioral interventions could modulate neural circuits that underlie addictive behaviors. Although this approach has only been explored in methods other than LIFU, combining its advantages over other neurostimulation methods with CBT could be revolutionary (156). This synergy could enhance the brain’s receptivity to therapy, potentially leading to more durable treatment outcomes and reduced relapse rates. This method of drug delivery facilitates delivery to target locations and overcomes blood-brain barrier permeability. This combination holds immense potential across various medical domains, including neurological disorders, cancer treatment, and gene therapy. By enabling targeted drug delivery while potentially reducing side effects, this approach offers a promising avenue for improved treatment paradigms. While still in the early stages of development, ongoing research aims to validate the efficacy of this approach through clinical trials, paving the way for its widespread adoption and transformative impact on patient care. The precise mechanisms by which LIFU affects neural activity and behavior are still under investigation, necessitating further research to optimize its use in conjunction with behavioral therapies.

Future research directions include pairing LIFU with real-time brain monitoring techniques, such as fMRI, to tailor treatments to individual neural activity patterns. This personalized approach could allow for the adjustment of LIFU parameters during treatment, potentially improving efficacy and patient outcomes. Studies investigating the integration of LIFU with monitoring technologies are essential to develop protocols that can adapt to the dynamic nature of brain activity during SUD treatment. Integrating LIFU into the multi-modal treatment landscape for SUD holds excellent promise. By combining this innovative technique with established pharmacological and behavioral interventions and harnessing the power of real-time brain imaging, researchers aim to create more effective and personalized treatment strategies. As evidence grows, LIFU could become a key component in the comprehensive treatment of SUDs, offering new hope to those affected. Rigorous clinical trials and continued scientific inquiry are vital to unlocking LIFU’s full potential and ensuring its safe and effective application in clinical settings (157).

## 7 Conclusion

LIFU presents a promising approach to SUD treatment, offering targeted neuromodulation without ablating brain tissue. Despite its advantages, the long-term effects of LIFU on brain tissue require exploration, necessitating global safety standards due to regional variations in guidelines. LIFU studies on SUD predominantly focus on the Nucleus Accumbens because of the complexity of SUD pathology. Therefore, further investigation should include other regions like the Insula and Amygdala. Optimization of LIFU parameters, including intensity and frequency, is ongoing to achieve desired outcomes with minimal adverse effects. The duality of LIFU effects underscores the complexity of neural modulation, warranting further exploration into underlying mechanisms. At the same time, integrating microbubbles in LIFU therapy shows promise for enhancing treatment efficacy. Large-scale studies with extended follow-up and robust controls are imperative to evaluate LIFU efficacy and safety compared to existing modalities. Personalized LIFU protocols based on neuroimaging data hold significant promise but require careful consideration of ethical implications. In summary, while LIFU offers a precise, noninvasive, and personalized therapeutic approach for SUD, its clinical realization depends on understanding mechanisms of action, optimizing protocols, and rigorous evaluation of efficacy and safety through interdisciplinary efforts.

## References

[1] World Drug Report. 35 million people worldwide suffer from drug use disorders while only 1 in 7 people receive treatment(2019). Available online at: www.unodc.org/unodc/en/frontpage/2019/June/world-drug-report-2019_-35-million-people-worldwide-suffer-from-drug-use-disorders-while-only-1-in-7-people-receive-treatment.html. (accessed March 13, 2024).

[2] Bewley-Taylor DR Nougier M. Measuring the ‘World drug problem’: 2019 and beyond. In: Klein A Stothard B, editors. Collapse Of The Global Order On Drugs: From UNGASS 2016 to review 2019. Leeds, England, United Kingdom: Emerald Publishing Limited (2018). p. 65–83. doi: 10.1108/978-1-78756-487-920181003

[3] Substance Abuse and Mental Health Services Administration. Key substance use and mental health indicators in the United States: Results from the 2021 National Survey on Drug Use and Health (HHS Publication No. PEP22-07-01-005, NSDUH Series H-57). Center for Behavioral Health Statistics and Quality, Substance Abuse and Mental Health Services Administration. (2022). Available online at: https://www.samhsa.gov/data/report/2021-nsduh-annual-national-report.

[4] Volkow ND Blanco C. Substance use disorders: a comprehensive update of classification, epidemiology, neurobiology, clinical aspects, treatment and prevention. World Psychiatry. (2023) 22:203–29. doi: 10.1002/wps.21073 PMC10168177 37159360

[5] D’Orsogna MR Böttcher L Chou T. Fentanyl-driven acceleration of racial, gender and geographical disparities in drug overdose deaths in the United States. PLoS Glob Public Health. (2023) 3:e0000769. doi: 10.1371/journal.pgph.0000769 36962959 PMC10032521

[6] Jones CM Bekheet F Park JN Alexander GC. The evolving overdose epidemic: synthetic opioids and rising stimulant-related harms. Epidemiol. Rev. (2020) 42:154–66. doi: 10.1093/epirev/mxaa011 PMC9200066 33511987

[7] Aletraris L Graves BD Ndung’u JJ. Assessing the impact of recreational cannabis legalization on cannabis use disorder and admissions to treatment in the United States. Curr Addict. Rep. (2023) 10:198–209. doi: 10.1007/s40429-023-00470-x 37266190 PMC10088679

[8] Nehring SM Chen RJ Freeman AM. Alcohol use disorder. In: StatPearls. StatPearls Publishing, Treasure Island (FL (2024).

[9] Hersey M Bacon AK Bailey LG Coggiano MA Newman AH Leggio L. Psychostimulant use disorder, an unmet therapeutic goal: can modafinil narrow the gap? Front Neurosci. (2021) 15:656475. doi: 10.3389/fnins.2021.656475 34121988 PMC8187604

[10] Rosenthal A Ebrahimi C Wedemeyer F Romanczuk-Seiferth N Beck A. The treatment of substance use disorders: recent developments and new perspectives. Neuropsychobiology. (2022) 81:451–72. doi: 10.1159/000525268 35724634

[11] Hser Y-I Saxon AJ Huang D Hasson A Thomas C Hillhouse M. Treatment retention among patients randomized to buprenorphine/naloxone compared to methadone in a multi-site trial. Addict. Abingdon Engl. (2014) 109:79–87. doi: 10.1111/add.12333 PMC3947022 23961726

[12] Principles of Drug Addiction Treatment: A Research-Based Guide (2012). Available online at: https://doi.apa.org/doi/10.1037/e686332012-001. (accessed March 13, 2024).

[13] Volkow ND Michaelides M Baler R. The neuroscience of drug reward and addiction. Physiol Rev. (2019) 99:2115–40. doi: 10.1152/physrev.00014.2018 PMC6890985 31507244

[14] Kalivas PW Volkow ND. New medications for drug addiction hiding in glutamatergic neuroplasticity. Mol Psychiatry. (2011) 16:974–86. doi: 10.1038/mp.2011.46 PMC3192324 21519339

[15] Di Chiara G Bassareo V. Reward system and addiction: what dopamine does and doesn’t do. Curr Opin Pharmacol. (2007) 7:69–76. doi: 10.1016/j.coph.2007.02.001 17174602

[16] Torregrossa MM Tang X-C Kalivas PW. The glutamatergic projection from the prefrontal cortex to the nucleus accumbens core is required for cocaine-induced decreases in ventral pallidal GABA. Neurosci Lett. (2008) 438:142–5. doi: 10.1016/j.neulet.2008.04.016 PMC2659572 18455875

[17] Quiroz C Orrú M Rea W Ciudad-Roberts A Yepes G Britt JP. Local control of extracellular dopamine levels in the medial nucleus accumbens by a glutamatergic projection from the infralimbic cortex. J Neurosci. (2016) 36:851–9. doi: 10.1523/jneurosci.2850-15.2016 PMC4719020 26791215

[18] Lovinger DM Gremel CM. A circuit-based information approach to substance abuse research. Trends Neurosci. (2021) 44:122–35. doi: 10.1016/j.tins.2020.10.005 PMC7856012 33168235

[19] Wilcox CE Pommy JM Adinoff B. Neural circuitry of impaired emotion regulation in substance use disorders. Am J Psychiatry. (2016) 173:344–61. doi: 10.1176/appi.ajp.2015.15060710 PMC4979988 26771738

[20] Sparing R Mottaghy FM. Noninvasive brain stimulation with transcranial magnetic or direct current stimulation (TMS/tDCS)—From insights into human memory to therapy of its dysfunction. Methods. (2008) 44:329–37. doi: 10.1016/j.ymeth.2007.02.001 18374276

[21] Deng Z-D Lisanby SH Peterchev AV. Electric field depth–focality tradeoff in transcranial magnetic stimulation: simulation comparison of 50 coil designs. Brain Stimulat. (2013) 6:1–13. doi: 10.1016/j.brs.2012.02.005 PMC3568257 22483681

[22] Opitz A Paulus W Will S Antunes A Thielscher A. Determinants of the electric field during transcranial direct current stimulation. NeuroImage. (2015) 109:140–50. doi: 10.1016/j.neuroimage.2015.01.033 25613437

[23] Spagnolo PA Goldman D. Neuromodulation interventions for addictive disorders: challenges, promise, and roadmap for future research. Brain J Neurol. (2017) 140:1183–203. doi: 10.1093/brain/aww284 PMC6059187 28082299

[24] Friedman D Johnson R Jr. Event-related potential (ERP) studies of memory encoding and retrieval: A selective review. Microsc. Res Tech. (2000) 51:6–28. doi: 10.1002/1097-0029(20001001)51:1<6::aid-jemt2>3.3.co;2-i 11002349

[25] Baek H Lockwood D Mason EJ Obusez E Poturalski M Rammo R. Clinical intervention using focused ultrasound (FUS) stimulation of the brain in diverse neurological disorders. Front Neurol. (2022) 13. doi: 10.3389/fneur.2022.880814 PMC9124976 35614924

[26] Koob GF Volkow ND. Neurobiology of addiction: a neurocircuitry analysis. Lancet Psychiatry. (2016) 3:760–73. doi: 10.1016/s2215-0366(16)00104-8 PMC6135092 27475769

[27] Verdejo-Garcia A Clark L Dunn BD. The role of interoception in addiction: A critical review. Neurosci Biobehav Rev. (2012) 36:1857–69. doi: 10.1016/j.neubiorev.2012.05.007 22659642

[28] Koob G. Addiction is a reward deficit and stress surfeit disorder. Front Psychiatry. (2013) 4. doi: 10.3389/fpsyt.2013.00072 PMC3730086 23914176

[29] Volkow ND Wang G-J Fowler JS Tomasi D Telang F. Addiction: Beyond dopamine reward circuitry. Proc Natl Acad Sci. (2011) 108:15037–42. doi: 10.1073/pnas.1010654108 PMC3174598 21402948

[30] Zhou K Xu H Lu S Jiang S Hou G Deng X. Reward and aversion processing by input-defined parallel nucleus accumbens circuits in mice. Nat Commun. (2022) 13:6244. doi: 10.1038/s41467-022-33843-3 36271048 PMC9587247

[31] Arco AD Mora F. Neurotransmitters and prefrontal cortex–limbic system interactions: implications for plasticity and psychiatric disorders. J Neural Transm. (2009) 116:941–52. doi: 10.1007/s00702-009-0243-8 19475335

[32] Lueptow LM Shashkova EC Miller MG Evans CJ Cahill CM. Insights into the neurobiology of craving in opioid use disorder. Curr Anesthesiol. Rep. (2020) 10:378–87. doi: 10.1007/s40140-020-00420-7 PMC7790122 33424457

[33] Fang Y Sun Y Liu Y Liu T Hao W Liao Y. Neurobiological mechanisms and related clinical treatment of addiction: a review. Psychoradiology. (2022) 2:180–9. doi: 10.1093/psyrad/kkac021 PMC10917179 38665277

[34] Vafaie N Kober H. Association of drug cues and craving with drug use and relapse: A systematic review and meta-analysis. JAMA Psychiatry. (2022) 79:641–50. doi: 10.1001/jamapsychiatry.2022.1240 PMC9161117 35648415

[35] Jackson ME Moghaddam B. Amygdala regulation of nucleus accumbens dopamine output is governed by the prefrontal cortex. J Neurosci. (2001) 21:676–81. doi: 10.1523/jneurosci.21-02-00676.2001 PMC6763812 11160446

[36] Stuber GD Sparta DR Stamatakis AM Van Leeuwen WA Hardjoprajitno J.E Cho S. Excitatory transmission from the amygdala to nucleus accumbens facilitates reward seeking. Nature. (2011) 475:377–80. doi: 10.1038/nature10194 PMC3775282 21716290

[37] Lammel S Lim BK Ran C Huang KW Betley MJ Tye K. Input-specific control of reward and aversion in the ventral tegmental area. Nature. (2012) 491:212–7. doi: 10.1038/nature11527 PMC3493743 23064228

[38] Chater TE Goda Y. The role of AMPA receptors in postsynaptic mechanisms of synaptic plasticity. Front Cell Neurosci. (2014) 8. doi: 10.3389/fncel.2014.00401 PMC4245900 25505875

[39] Stamatakis AM Sparta DR Jennings JH McElligott ZA Decot H Stuber GD. Amygdala and Bed Nucleus of the Stria Terminalis Circuitry: Implications for addiction-related behaviors. Neuropharmacology. (2014) 76:320–8. doi: 10.1016/j.neuropharm.2013.05.046 PMC3858407 23752096

[40] Bouarab C Thompson B Polter AM. VTA GABA neurons at the interface of stress and reward. Front Neural Circuits. (2019) 13:78. doi: 10.3389/fncir.2019.00078 31866835 PMC6906177

[41] Aragona BJ Cleaveland NA Stuber GD Day JJ Carelli RM Wightman RM. Preferential enhancement of dopamine transmission within the nucleus accumbens shell by cocaine is attributable to a direct increase in phasic dopamine release events. J Neurosci. (2008) 28:8821–31. doi: 10.1523/jneurosci.2225-08.2008 PMC2584805 18753384

[42] Wright AL Vissel B. The essential role of AMPA receptor GluR2 subunit RNA editing in the normal and diseased brain. Front Mol Neurosci. (2012) 5. doi: 10.3389/fnmol.2012.00034 PMC3324117 22514516

[43] Liechti ME Lhuillier L Kaupmann K Markou A. Metabotropic glutamate 2/3 receptors in the ventral tegmental area and the nucleus accumbens shell are involved in behaviors relating to nicotine dependence. J Neurosci. (2007) 27:9077–85. doi: 10.1523/jneurosci.1766-07.2007 PMC6672208 17715344

[44] Chen Y-W Lin H-C Ng M-C Hsiao Y-H Wang C-C Gean P-W Chen PS. Activation of mGluR2/3 underlies the effects of N-acetylcystein on amygdala-associated autism-like phenotypes in a valproate-induced rat model of autism. Front Behav Neurosci. (2014) 8. doi: 10.3389/fnbeh.2014.00219 PMC4060031 24987341

[45] Di Chiara G Bassareo V Fenu S De Luca MA Spina L Cadoni C. Dopamine and drug addiction: the nucleus accumbens shell connection. Neuropharmacology. (2004) 47:227–41. doi: 10.1016/j.neuropharm.2004.06.032 15464140

[46] Moorman DE Aston-Jones G. Prelimbic and infralimbic medial prefrontal cortex neuron activity signals cocaine seeking variables across multiple timescales. Psychopharmacol (Berl.). (2023) 240:575–94. doi: 10.1007/s00213-022-06287-2 PMC10406502 36464693

[47] Shin CB Templeton TJ Chiu AS Kim J Gable ES Vieira PA. Endogenous glutamate within the prelimbic and infralimbic cortices regulates the incubation of cocaine-seeking in rats. Neuropharmacology. (2018) 128:293–300. doi: 10.1016/j.neuropharm.2017.10.024 29061508 PMC6400061

[48] McFarland K Lapish CC Kalivas PW. Prefrontal glutamate release into the core of the nucleus accumbens mediates cocaine-induced reinstatement of drug-seeking behavior. J Neurosci. (2003) 23:3531–7. doi: 10.1523/jneurosci.23-08-03531.2003 PMC6742291 12716962

[49] McLaughlin J See RE. Selective inactivation of the dorsomedial prefrontal cortex and the basolateral amygdala attenuates conditioned-cued reinstatement of extinguished cocaine-seeking behavior in rats. Psychopharmacol (Berl.). (2003) 168:57–65. doi: 10.1007/s00213-002-1196-x 12845418

[50] Berglind WJ Whitfield TW LaLumiere RT Kalivas PW McGinty JF. A single intra-PFC infusion of BDNF prevents cocaine-induced alterations in extracellular glutamate within the nucleus accumbens. J Neurosci. (2009) 29:3715–9. doi: 10.1523/jneurosci.5457-08.2009 PMC2683065 19321768

[51] McGinty JF Zelek-Molik A Sun W-L. Cocaine self-administration causes signaling deficits in corticostriatal circuitry that are reversed by BDNF in early withdrawal. Brain Res. (2015) 1628:82–7. doi: 10.1016/j.brainres.2014.09.050 PMC4377116 25268928

[52] Ruisoto P Contador I. The role of stress in drug addiction. An integrative review. Physiol Behav. (2019) 202:62–8. doi: 10.1016/j.physbeh.2019.01.022 30711532

[53] Sharp BM. Basolateral amygdala and stress-induced hyperexcitability affect motivated behaviors and addiction. Transl Psychiatry. (2017) 7:e1194. doi: 10.1038/tp.2017.161 28786979 PMC5611728

[54] Koya E Spijker S Voorn P Binnekade R Schmidt ED Schoffelmeer ANM. Enhanced cortical and accumbal molecular reactivity associated with conditioned heroin, but not sucrose-seeking behaviour. J Neurochem. (2006) 98:905–15. doi: 10.1111/j.1471-4159.2006.03917.x 16787418

[55] Jin Z Bhandage AK Bazov I Kononenko O Bakalkin G Korpi ER. Expression of specific ionotropic glutamate and GABA-A receptor subunits is decreased in central amygdala of alcoholics. Front Cell Neurosci. (2014) 8:288. doi: 10.3389/fncel.2014.00288 25278838 PMC4165314

[56] Cain ME Denehy ER Bardo MT. Individual differences in amphetamine self-administration: the role of the central nucleus of the amygdala. Neuropsychopharmacology. (2008) 33:1149–61. doi: 10.1038/sj.npp.1301478 PMC2742632 17568395

[57] Lu L Dempsey J Shaham Y Hope BT. Differential long-term neuroadaptations of glutamate receptors in the basolateral and central amygdala after withdrawal from cocaine self-administration in rats. J Neurochem. (2005) 94:161–8. doi: 10.1111/j.1471-4159.2005.03178.x 15953359

[58] Young KA Gobrogge KL Wang Z. The role of mesocorticolimbic dopamine in regulating interactions between drugs of abuse and social behavior. Neurosci Biobehav Rev. (2011) 35:498–515. doi: 10.1016/j.neubiorev.2010.06.004 20600286 PMC3578706

[59] Willuhn I Wanat MJ Clark JJ Phillips PEM. Dopamine signaling in the nucleus accumbens of animals self-administering drugs of abuse. Curr Top Behav Neurosci. (2010) 3:29–71. doi: 10.1007/7854_2009_27 21161749 PMC3766749

[60] Zinsmaier AK Dong Y Huang YH. Cocaine-induced projection-specific and cell type-specific adaptations in the nucleus accumbens. Mol Psychiatry. (2022) 27:669–86. doi: 10.1038/s41380-021-01112-2 PMC8691189 33963288

[61] Perreault ML Fan T Alijaniaram M O’Dowd BF George SR. Dopamine D1–D2 receptor heteromer in dual phenotype GABA/glutamate-coexpressing striatal medium spiny neurons: regulation of BDNF, GAD67 and VGLUT1/2. PLoS One. (2012) 7:e33348. doi: 10.1371/journal.pone.0033348 22428025 PMC3299775

[62] Lazzaretti M Mandolini GM Altamura AC Brambilla P. Substances of abuse and hallucinogenic activity: the dopaminergic pathway - focus on cocaine and amphetamine-type stimulants. In: Brambilla P Mauri MC Altamura AC, editors. Hallucinations in Psychoses and Affective Disorders: A Clinical and Biological Approach. Springer International Publishing, Cham (2018). p. 3–16. doi: 10.1007/978-3-319-75124-5_1

[63] Parsegian A See RE. Dysregulation of dopamine and glutamate release in the prefrontal cortex and nucleus accumbens following methamphetamine self-administration and during reinstatement in rats. Neuropsychopharmacology. (2014) 39:811–22. doi: 10.1038/npp.2013.231 PMC3924513 23995583

[64] McGlinchey EM James MH Mahler SV Pantazis C Aston-Jones G. Prelimbic to accumbens core pathway is recruited in a dopamine-dependent manner to drive cued reinstatement of cocaine seeking. J Neurosci Off J Soc Neurosci. (2016) 36:8700–11. doi: 10.1523/jneurosci.1291-15.2016 PMC4987439 27535915

[65] Struik RF Marchant NJ de Haan R Terra H van Mourik Y Schetters D. Dorsomedial prefrontal cortex neurons encode nicotine-cue associations. Neuropsychopharmacology. (2019) 44:2011–21. doi: 10.1038/s41386-019-0449-x PMC6898138 31242502

[66] See RE Fuchs RA Ledford CC McLAUGHLIN J. Drug addiction, relapse, and the amygdala. Ann N Y. Acad Sci. (2003) 985:294–307. doi: 10.1111/j.1749-6632.2003.tb07089.x 12724166

[67] Crunelle CL Kaag AM van den Munkhof HE Reneman L Homberg JR Sabbe B. Dysfunctional amygdala activation and connectivity with the prefrontal cortex in current cocaine users. Hum Brain Mapp. (2015) 36:4222–30. doi: 10.1002/hbm.22913 PMC6869379 26220024

[68] Li X Zeric T Kambhampati S Bossert JM Shaham Y. The central amygdala nucleus is critical for incubation of methamphetamine craving. Neuropsychopharmacology. (2015) 40:1297–306. doi: 10.1038/npp.2014.320 PMC4367476 25475163

[69] Fields HL Margolis EB. Understanding opioid reward. Trends Neurosci. (2015) 38:217–25. doi: 10.1016/j.tins.2015.01.002 PMC4385443 25637939

[70] Morel C Montgomery S Han M-H. Nicotine and alcohol: the role of midbrain dopaminergic neurons in drug reinforcement. Eur J Neurosci. (2019) 50:2180–200. doi: 10.1111/ejn.14160 PMC6431587 30251377

[71] Hwa L Besheer J Kash T. Glutamate plasticity woven through the progression to alcohol use disorder: a multi-circuit perspective. F1000Research. (2017) 6:298. doi: 10.12688/f1000research.9609.1 28413623 PMC5365217

[72] Shnitko TA Robinson DL. Regional variation in phasic dopamine release during alcohol and sucrose self-administration in rats. ACS Chem Neurosci. (2015) 6:147–54. doi: 10.1021/cn500251j PMC4304482 25493956

[73] Murphy A Taylor E Elliott R. The detrimental effects of emotional process dysregulation on decision-making in substance dependence. Front Integr Neurosci. (2012) 6:101. doi: 10.3389/fnint.2012.00101 23162443 PMC3491319

[74] Wise RA Koob GF. The development and maintenance of drug addiction. Neuropsychopharmacology. (2014) 39:254–62. doi: 10.1038/npp.2013.261 PMC3870778 24121188

[75] Peña B. Prefrontal synaptic glutamate transmission dynamics across psychostimulants and behavioral paradigms of drug addiction. MUSC Theses Diss. (2017).

[76] Arce C Mira RG Lira M Cerpa W. Binge-like alcohol administration alters decision making in an adolescent rat model: role of N-methyl-D-aspartate receptor signaling. Stresses. (2024) 4:1–13. doi: 10.3390/stresses4010001

[77] Naqvi NH Bechara A. The hidden island of addiction: the insula. Trends Neurosci. (2009) 32:56–67. doi: 10.1016/j.tins.2008.09.009 18986715 PMC3698860

[78] Ghareh H Alonso-Lozares I Schetters D Herman RJ Heistek TS Van Mourik Y. Role of anterior insula cortex in context-induced relapse of nicotine-seeking. eLife. (2022) 11:e75609. doi: 10.7554/elife.75609 35536612 PMC9119676

[79] Cosme CV Gutman AL LaLumiere RT. The Dorsal Agranular Insular Cortex Regulates the Cued Reinstatement of Cocaine-Seeking, but not Food-Seeking, Behavior in Rats. Neuropsychopharmacology. (2015) 40:2425–33. doi: 10.1038/npp.2015.92 PMC4538357 25837282

[80] Janes AC Gilman JM Radoman M Pachas G Fava M Evins AE. Revisiting the role of the insula and smoking cue-reactivity in relapse: A replication and extension of neuroimaging findings. Drug Alcohol Depend. (2017) 179:8–12. doi: 10.1016/j.drugalcdep.2017.06.012 28735078 PMC5599349

[81] Vertes RP. Differential projections of the infralimbic and prelimbic cortex in the rat. Synap. N Y. N. (2004) 51:32–58. doi: 10.1002/syn.10279 14579424

[82] Devinsky O Morrell MJ Vogt BA. Contributions of anterior cingulate cortex to behaviour. Brain. (1995) 118:279–306. doi: 10.1093/brain/118.1.279 7895011

[83] Medford N Critchley HD. Conjoint activity of anterior insular and anterior cingulate cortex: awareness and response. Brain Struct Funct. (2010) 214:535–49. doi: 10.1007/s00429-010-0265-x PMC2886906 20512367

[84] Droutman V Bechara A Read SJ. Roles of the different sub-regions of the insular cortex in various phases of the decision-making process. Front Behav Neurosci. (2015) 9. doi: 10.3389/fnbeh.2015.00309 PMC4658437 26635559

[85] Venniro M Caprioli D Zhang M Whitaker LR Zhang S Warren BL. The anterior insular cortex→Central amygdala glutamatergic pathway is critical to relapse after contingency management. Neuron. (2017) 96:414–427.e8. doi: 10.1016/j.neuron.2017.09.024 29024664 PMC5687288

[86] Garland EL Froeliger B Zeidan F Partin K Howard MO. The downward spiral of chronic pain, prescription opioid misuse, and addiction: Cognitive, affective, and neuropsychopharmacologic pathways. Neurosci Biobehav Rev. (2013) 37:2597–607. doi: 10.1016/j.neubiorev.2013.08.006 PMC3967721 23988582

[87] Franklin TR Acton PD Maldjian JA Gray JD Croft JR Dackis CA. Decreased gray matter concentration in the insular, orbitofrontal, cingulate, and temporal cortices of cocaine patients. Biol Psychiatry. (2002) 51:134–42. doi: 10.1016/s0006-3223(01)01269-0 11822992

[88] Scala L Muscatello MRA Pangallo N Bruno A Zoccali RA. Neurobiological and psychopathological mechanisms underlyng addiction-like behaviors: an overview and thematic synthesis. Mediterr J Clin Psychol. (2017) 5(2). doi: /10.6092/2282-1619/2017.5.1626

[89] Duresso S. Psychopharmacological perspectives and diagnosis of substance use disorder. In: Addictions - Diagnosis and Treatment. London, Onited Kingdom: IntechOpen (2021). doi: 10.5772/intechopen.99531

[90] Alho E Cordeiro J Assumpcao de Monaco B Jagid J. Introduction and History of Neuromodulation for Pain. Cham, Switzerland: Springer (2022). pp. 1–21. doi: 10.1007/978-3-030-84778-4_1.

[91] Rezayat E Toostani IG. A review on brain stimulation using low intensity focused ultrasound. Basic Clin Neurosci. (2016) 7:187–94. doi: 10.15412/J.BCN.03070303 PMC4981830 27563411

[92] Dell’Italia J Sanguinetti JL Monti MM Bystritsky A Reggente N. Current state of potential mechanisms supporting low intensity focused ultrasound for neuromodulation. Front Hum Neurosci. (2022) 16. doi: 10.3389/fnhum.2022.872639 PMC9081930 35547195

[93] Fan B Goodman W Cho RY Sheth SA Bouchard RR Aazhang B. Computational modeling and minimization of unintended neuronal excitation in a LIFU stimulation. Sci Rep. (2023) 13:13403. doi: 10.1038/s41598-023-40522-w 37591991 PMC10435497

[94] Guerra A Bologna M. Low-intensity transcranial ultrasound stimulation: mechanisms of action and rationale for future applications in movement disorders. Brain Sci. (2022) 12:611. doi: 10.3390/brainsci12050611 35624998 PMC9139935

[95] Siebner HR Funke K Aberra AS Antal A Bestmann S Chen R. Transcranial magnetic stimulation of the brain: What is stimulated? – A consensus and critical position paper. Clin Neurophysiol. (2022) 140:59–97. doi: 10.1016/j.clinph.2022.04.022 35738037 PMC9753778

[96] Strangman GE Zhang Q Li Z. Scalp and skull influence on near infrared photon propagation in the Colin27 brain template. NeuroImage. (2014) 85:136–49. doi: 10.1016/j.neuroimage.2013.04.090 23660029

[97] Aubry J-F Attali D Schafer M Fouragnan E Caskey C Chen R. ITRUSST consensus on biophysical safety for transcranial ultrasonic stimulation. arXiv. (2024). doi: 10.48550/arXiv.2311.05359 PMC12644226 41072763

[98] Wang Y Guo L. Nanomaterial-enabled neural stimulation. Front Neurosci. (2016) 10. doi: 10.3389/fnins.2016.00069 PMC4779906 27013938

[99] Kim CK Adhikari A Deisseroth K. Integration of optogenetics with complementary methodologies in systems neuroscience. Nat Rev Neurosci. (2017) 18:222–35. doi: 10.1038/nrn.2017.15 PMC5708544 28303019

[100] Kubanek J Shukla P Das A Baccus SA Goodman MB. Ultrasound elicits behavioral responses through mechanical effects on neurons and ion channels in a simple nervous system. J Neurosci. (2018) 38:3081–91. doi: 10.1523/jneurosci.1458-17.2018 PMC5864152 29463641

[101] Zhu J Xian Q Hou X Wong KF Zhu T Chen Z. The mechanosensitive ion channel Piezo1 contributes to ultrasound neuromodulation. Proc Natl Acad Sci. (2023) 120:e2300291120. doi: 10.1073/pnas.2300291120 37098060 PMC10161134

[102] Lim J Tai H-H Liao W-H Chu Y-C Hao C-M Huang Y-C. ASIC1a is required for neuronal activation via low-intensity ultrasound stimulation in mouse brain. eLife. (2021) 10:e61660. doi: 10.7554/elife.61660 34569932 PMC8510583

[103] Burks SR Lorsung RM Nagle ME Tu T-W Frank JA. Focused ultrasound activates voltage-gated calcium channels through depolarizing TRPC1 sodium currents in kidney and skeletal muscle. Theranostics. (2019) 9:5517–31. doi: 10.7150/thno.33876 PMC6735402 31534500

[104] Ranade SS Syeda R Patapoutian A. Mechanically activated ion channels. Neuron. (2015) 87:1162–79. doi: 10.1016/j.neuron.2015.08.032 PMC4582600 26402601

[105] Kubanek J Shi J Marsh J Chen D Deng C Cui J. Ultrasound modulates ion channel currents. Sci Rep. (2016) 6:24170. doi: 10.1038/srep24170 27112990 PMC4845013

[106] Ye J Tang S Meng L Li X Wen X Chen S. Ultrasonic control of neural activity through activation of the mechanosensitive channel mscL. Nano Lett. (2018) 18:4148–55. doi: 10.1021/acs.nanolett.8b00935 29916253

[107] Duque M Lee-Kubli CA Tufail Y Magaram U Patel J Chakraborty A. Sonogenetic control of mammalian cells using exogenous Transient Receptor Potential A1 channels. Nat Commun. (2022) 13:600. doi: 10.1038/s41467-022-28205-y 35140203 PMC8828769

[108] Mueller JK Tyler WJ. A quantitative overview of biophysical forces impinging on neural function. Phys Biol. (2014) 11:051001. doi: 10.1088/1478-3975/11/5/051001 25156965

[109] Taylor GJ Heberle FA Seinfeld JS Katsaras J Collier CP Sarles SA. Capacitive detection of low-enthalpy, higher-order phase transitions in synthetic and natural composition lipid membranes. Langmuir. (2017) 33:10016–26. doi: 10.1021/acs.langmuir.7b02022 28810118

[110] Ahmadpoor F Sharma P. Flexoelectricity in two-dimensional crystalline and biological membranes. Nanoscale. (2015) 7:16555–70. doi: 10.1039/c5nr04722f 26399878

[111] El Hady A Machta BB. Mechanical surface waves accompany action potential propagation. Nat Commun. (2015) 6:6697. doi: 10.1038/ncomms7697 25819404

[112] Jerusalem A Al-Rekabi Z Chen H Ercole A Malboubi M Tamayo-Elizalde M. Electrophysiological-mechanical coupling in the neuronal membrane and its role in ultrasound neuromodulation and general anaesthesia. Acta Biomater. (2019) 97:116–40. doi: 10.1016/j.actbio.2019.07.041 31357005

[113] Plaksin M Shoham S Kimmel E. Intramembrane cavitation as a predictive bio-piezoelectric mechanism for ultrasonic brain stimulation. Phys Rev X. (2014) 4:011004. doi: 10.1103/physrevx.4.011004

[114] Krasovitski B Frenkel V Shoham S Kimmel E. Intramembrane cavitation as a unifying mechanism for ultrasound-induced bioeffects. Proc Natl Acad Sci U. S. A. (2011) 108:3258–63. doi: 10.1073/pnas.1015771108 PMC3044354 21300891

[115] Plaksin M Kimmel E Shoham S. Cell-type-selective effects of intramembrane cavitation as a unifying theoretical framework for ultrasonic neuromodulation. eNeuro. (2016) 3. doi: 10.1523/eneuro.0136-15.2016 PMC4917736 27390775

[116] Hameroff S Trakas M Duffield C Annabi E Gerace MB Boyle P. Transcranial ultrasound (TUS) effects on mental states: A pilot study. Brain Stimulat. (2013) 6:409–15. doi: 10.1016/j.brs.2012.05.002 22664271

[117] Darrow DP O’Brien P Richner T Netoff TI Ebbini ES. A thermal mechanism underlies tFUS neuromodulation. Brain Stimulat. (2020) 13:327–8. doi: 10.1016/j.brs.2019.10.018 31812450

[118] Yoo S-S Bystritsky A Lee J-H Zhang Y Fischer K Min B-K. Focused ultrasound modulates region-specific brain activity. NeuroImage. (2011) 56:1267–75. doi: 10.1016/j.neuroimage.2011.02.058 PMC3342684 21354315

[119] Martin E Aubry J-F Schafer M Verhagen L Treeby B Pauly KB. ITRUSST consensus on standardised reporting for transcranial ultrasound stimulation. Brain Stimulat. (2024) 17:607–15. doi: 10.1016/j.brs.2024.04.013 PMC12436198 38670224

[120] Kelley P Whatson T. Making long-term memories in minutes: a spaced learning pattern from memory research in education. Front Hum Neurosci. (2013) 7. doi: 10.3389/fnhum.2013.00589 PMC3782739 24093012

[121] Bliss TVP Collingridge GL Morris RGM Morris RGM. Long-term potentiation and memory. Philos Trans R Soc Lond B Biol Sci. (2003) 358:643–7. doi: 10.1098/rstb.2002.1230 PMC1693171 12740109

[122] Olaitan GO Ganesana M Lynch WJ Legon W Venton BJ. Focused Ultrasound modulates dopamine in a mesolimbic reward circuit. bioRxiv. (2022) 2024.02.13.580202. doi: 10.1101/2024.02.13.580202 PMC11791541 39902479

[123] Zeng K Darmani G Fomenko A Xia X Tran S Nankoo J-F. Induction of Human Motor Cortex Plasticity by Theta Burst Transcranial Ultrasound Stimulation. Ann Neurol. (2022) 91:238–52. doi: 10.1002/ana.26294 34964172

[124] Deveci E Akbaş F Ergun AŞ Kurtulmuş A Koçak AB Boyraz RK. The effects of transcranial focused ultrasound stimulation of nucleus accumbens on neuronal gene expression and brain tissue in high alcohol-preferring rats. Mol Neurobiol. (2023) 60:1099–116. doi: 10.1007/s12035-022-03130-9 36417101

[125] Lan Y Feng Y Zheng Y Yang Z Zhao Y Zhu M. Effectiveness of low intensity focused ultrasound stimulation of nucleus accumbens in mice. kmykdxxb. (2021) 42:13–9. doi: 10.12259/j.issn.2095-610X.S20210703

[126] In A Strohman A Payne B Legon W. Low-intensity focused ultrasound to the insula and dorsal anterior cingulate has site-specific and pressure dependent effects on pain during measures of central sensitization. (2024) 01:10. doi: 10.1101/2023.05.05.539593

[127] Legon W Strohman A In A Payne B. Noninvasive neuromodulation of subregions of the human insula differentially affect pain processing and heart-rate variability: a within-subjects pseudo-randomized trial. PAIN. (2024) 165(7):1625–41. doi: 10.1097/j.pain.0000000000003171 PMC11189760 38314779

[128] Kim MG Yu K Yeh C-Y Fouda R Argueta D Kiven S. Low-intensity transcranial focused ultrasound suppresses pain by modulating pain processing brain circuits. (2022) 12:2022.12.07.519518. doi: 10.1101/2022.12.07.519518 PMC11406192 38976875

[129] Niu X Yu K He B. Transcranial focused ultrasound induces sustained synaptic plasticity in rat hippocampus. Brain Stimulation. (2022) 15(2):352–9. 10.1016/j.brs.2022.01.015 PMC9295311 35104664

[130] Niu L Guo Y Lin Z Shi Z Bian T Qi L. Noninvasive ultrasound deep brain stimulation of nucleus accumbens induces behavioral avoidance. Sci China Life Sci. (2020) 63:1328–36. doi: 10.1007/s11427-019-1616-6 32180109

[131] Wang Y Bai Y Xiao X Wang L Wei G Guo M. Low-intensity focused ultrasound stimulation reverses social avoidance behavior in mice experiencing social defeat stress. Cereb Cortex. (2022) 32:5580–96. doi: 10.1093/cercor/bhac037 35188969

[132] Yi S Zou J Meng L Chen H Hong Z Liu X. Ultrasound stimulation of prefrontal cortex improves lipopolysaccharide-induced depressive-like behaviors in mice. Front Psychiatry. (2022) 13. doi: 10.3389/fpsyt.2022.864481 PMC9099414 35573384

[133] Ren S Guo Z Zhang J He Y Sun Z Yang J. Transcriptomic alterations in the hippocampus and prefrontal cortex of chronic unpredictable stress rats induced by low-intensity pulsed ultrasound. (2024). doi: 10.21203/rs.3.rs-3775894/v1 PMC11953113 39663283

[134] Kim YG Kim SE Lee J Hwang S Yoo S-S Lee HW. Neuromodulation using transcranial focused ultrasound on the bilateral medial prefrontal cortex. J Clin Med. (2022) 11:3809. doi: 10.3390/jcm11133809 35807094 PMC9267901

[135] Huang X Zheng H Lin Z Wang K Liu X Zhou W. Transcranial low-intensity pulsed ultrasound modulates structural and functional synaptic plasticity in rat hippocampus. IEEE Trans Ultrason. Ferroelectr. Freq. Control. (2019) 66:930–8. doi: 10.1109/tuffc.2019.2903896 30869615

[136] Pan T-Y Pan Y-J Tsai S-J Tsai C-W Yang F-Y. Focused ultrasound stimulates the prefrontal cortex and prevents MK-801-induced psychiatric symptoms of schizophrenia in rats. Schizophr. Bull. (2024) 50:120–31. doi: 10.1093/schbul/sbad078 PMC10754174 37301986

[137] Xie Z Dong S Zhang Y Yuan Y. Transcranial ultrasound stimulation at the peak-phase of theta-cycles in the hippocampus improve memory performance. NeuroImage. (2023) 283:120423. doi: 10.1016/j.neuroimage.2023.120423 37884166

[138] Chou T Deckersbach T Guerin B Sretavan Wong K Borron BM Kanabar A. Transcranial focused ultrasound of the amygdala modulates fear network activation and connectivity. Brain Stimulat. (2024) 17:312–20. doi: 10.1016/j.brs.2024.03.004 38447773

[139] Kuhn T Spivak NM Dang BH Becerra S Halavi SE Rotstein N. Transcranial focused ultrasound selectively increases perfusion and modulates functional connectivity of deep brain regions in humans. Front Neural Circuits. (2023) 17. doi: 10.3389/fncir.2023.1120410 PMC10114286 37091318

[140] Folloni D Verhagen L Mars RB Fouragnan E Constans C Aubry J-F. Manipulation of subcortical and deep cortical activity in the primate brain using transcranial focused ultrasound stimulation. Neuron. (2019) 101:1109–1116.e5. doi: 10.1016/j.neuron.2019.01.019 30765166 PMC6520498

[141] Cole SR Voytek B. Brain oscillations and the importance of waveform shape. Trends Cogn. Sci. (2017) 21:137–49. doi: 10.1016/j.tics.2016.12.008 28063662

[142] Klimesch W. The frequency architecture of brain and brain body oscillations: an analysis. Eur J Neurosci. (2018) 48:2431–53. doi: 10.1111/ejn.14192 PMC6668003 30281858

[143] Klimesch W. Memory processes, brain oscillations and EEG synchronization. Int J Psychophysiol. (1996) 24:61–100. doi: 10.1016/s0167-8760(96)00057-8 8978436

[144] Howard MW Rizzuto DS Caplan JB Madsen JR Lisman J Aschenbrenner-Scheibe R. Gamma oscillations correlate with working memory load in humans. Cereb Cortex. (2003) 13:1369–74. doi: 10.1093/cercor/bhg084 14615302

[145] Sharott A Magill PJ Harnack D Kupsch A Meissner W Brown P. Dopamine depletion increases the power and coherence of β-oscillations in the cerebral cortex and subthalamic nucleus of the awake rat. Eur J Neurosci. (2005) 21:1413–22. doi: 10.1111/j.1460-9568.2005.03973.x 15813951

[146] Mallet N Pogosyan A Sharott A Csicsvari J Bolam J Brown P. Disrupted dopamine transmission and the emergence of exaggerated beta oscillations in subthalamic nucleus and cerebral cortex. J Neurosci Off J Soc Neurosci. (2008) 28:4795–806. doi: 10.1523/jneurosci.0123-08.2008 PMC6670450 18448656

[147] Mahoney JJ Haut MW Carpenter J Ranjan M Thompson-Lake DGY Marton JL. Low-intensity focused ultrasound targeting the nucleus accumbens as a potential treatment for substance use disorder: safety and feasibility clinical trial. Front Psychiatry. (2023) 14. doi: 10.3389/fpsyt.2023.1211566 PMC10540197 37779628

[148] Lin C-W Cheng M-H Fan C-H Chen H-H Yeh C-K. Focused ultrasound stimulation of infralimbic cortex attenuates reinstatement of methamphetamine-induced conditioned place preference in rats. Neurother. J Am Soc Exp Neurother. (2024) 21:e00328. doi: 10.1016/j.neurot.2024.e00328 PMC10937235 38355360

[149] Wang F Cai Q Ju R Wang S Liu L Pan M. Low-intensity focused ultrasound ameliorates depression-like behaviors associated with improving the synaptic plasticity in the vCA1-mPFC pathway. Cereb Cortex. (2023) 33:8024–34. doi: 10.1093/cercor/bhad095 37041107

[150] Baek H Pahk KJ Kim H. A review of low-intensity focused ultrasound for neuromodulation. Biomed Eng. Lett. (2017) 7:135–42. doi: 10.1007/s13534-016-0007-y PMC6208465 30603160

[151] Mahoney JJ Thompson-Lake DGY Ranjan M Marton JL Carpenter JS Zheng W. Low-intensity focused ultrasound targeting the bilateral nucleus accumbens as a potential treatment for substance use disorder: A first-in-human report. Biol Psychiatry. (2023) 94:e41–3. doi: 10.1016/j.biopsych.2023.06.031 37610405

[152] Peng X Connolly DJ Sutton F Robinson J Baker-Vogel B Short EB. Non-invasive suppression of the human nucleus accumbens (NAc) with transcranial focused ultrasound (tFUS) modulates the reward network: a pilot study. Front Hum Neurosci. (2024) 18. doi: 10.3389/fnhum.2024.1359396 PMC11018963 38628972

[153] Wang B Chen M-X Chen S-C Feng X-J Liao Y-H Zhao Y-X. Low-intensity focused ultrasound alleviates chronic neuropathic pain-induced allodynia by inhibiting neuroplasticity in the anterior cingulate cortex. Neural Plast. (2022) 2022:e6472475. doi: 10.1155/2022/6472475 PMC9338851 35915650

[154] Rezai A. A randomized, sham-controlled trial investigating low intensity focused ultrasound as a novel treatment for refractory opioid use disorder(2024). Available online at: https://clinicaltrials.gov/study/NCT06218706. (accessed December, 23, 2023).

[155] InSightec. A Feasibility Clinical Trial of Exablate for Low Intensity Focused Ultrasound Neuromodulation in Patients With Opioid Use Disorder (OUD) and/or Other Substance Abuse Disorders (SUDs) (2024). Available online at: https://clinicaltrials.gov/study/NCT04197921. (accessed December, 23, 2023).

[156] Richter K Peter L Acker J Höfig J Miloseva L Niklewski G. Personalized approach in the treatment of tinnitus and insomnia: combining repetitive transcranial magnetic stimulation and cognitive behavioral therapy. EPMA J Suppl. (2017) 8:1–54. doi: 10.1007/s13167-017-0108-4 PMC5399016 28484405

[157] Xu B LaBar KS. Advances in understanding addiction treatment and recovery. Sci Adv. (2019) 5:eaaz6596. doi: 10.1126/sciadv.aaz6596 31663030 PMC6795505

